# Supplementary material for: Anaerobic bacteria Cetobacterium sp. nov C33 plays a crucial role in the intestinal microbial balance and regulation of gene expression to immune and metabolic responses in Nile tilapia
Source: PLoS One. 2026 May 7;21(5):e0344851. doi: 10.1371/journal.pone.0344851 (PMC13152124; doi:10.1371/journal.pone.0344851)
Supplement: S1 Table — Differentially up-regulated genes from Oreochromis niloticus following Cetobacterium sp. nov C33 (p < 0.05), in kidney samples of tilapia fingerlings from the group fed with the Cetobacterium diet (C33D): basal diet with 1 x 10^8^ CFU/g of Cetobacterium sp. nov C33, compared to the control group fed with Control Diet (CD): basal diet; after ceasing consumption of Cetobacterium Diet for 5 days. (DOCX) [file pone.0344851.s001.docx]

S1 Table. Differentially up-regulated genes.

Differentially up-regulated genes from *Oreochromis niloticus* following *Cetobacterium* sp. nov C33 (*p*<0.05), in kidney samples of tilapia fingerlings from the group fed with the *Cetobacterium* diet (C33D): basal diet with 1 x 10^8^ CFU/g of *Cetobacterium* sp. nov C33, compared to the control group fed with Control Diet (CD): basal diet; after ceasing consumption of *Cetobacterium* Diet for 5 days.

| Symbol | Description |
| --- | --- |
| LOC109204053 | Hemicentin-2 |
| LOC100705032 | Transcription factor BTF3 homolog 4 |
| trpm5 | Transient receptor potential cation channel subfamily M member 5 |
| LOC102077504 | LRR and PYD domains-containing protein 3 |
| LOC102083021 | Uncharacterized LOC102083021 |
| LOC112845103 | Uncharacterized LOC112845103 |
| nkx6-3 | Homeobox protein Nkx-6.3 |
| LOC112846614 | Uncharacterized LOC112846614 |
| LOC109204180 | C-reactive protein |
| tmc4 | Transmembrane channel-like 4 |
| mrps18c | Mitochondrial ribosomal protein S18C |
| LOC102077416 | Uncharacterized LOC102077416 |
| glod5 | Glyoxalase domain containing 5 |
| LOC100699319 | Somatostatin receptor type 5 |
| LOC109198880 | Uncharacterized LOC109198880 |
| rcn1 | Beta-1,2-xylosyltransferase RCN11 |
| LOC100699084 | Uncharacterized protein LOC100699084 isoform X2 |
| ano9 | Anoctamin 9b |
| LOC112844035 | 5S ribosomal RNA |
| LOC100705987 | NADPH oxidase organizer 1 |
| LOC100694215 | Dehydrogenase/reductase SDR family member 11 |
| ndufa2 | NADH dehydrogenase (ubiquinone) 1 alpha subcomplex subunit 2 |
| LOC109194889 | Sodium channel subunit beta-3 |
| LOC106098049 | Alpha-(1,3)-fucosyltransferase 9 |
| LOC112845751 | 5.8S ribosomal RNA |
| LOC100710604 | Beta-galactoside-binding lectin |
| LOC112843554 | Class I histocompatibility antigen, F10 alpha chain-like |
| pln | 50S ribosomal protein L14 |
| LOC100696284 | Protein S100-A1 |
| LOC102083293 | Uncharacterized LOC102083293 |
| LOC109200871 | Cystatin-B |
| LOC100705610 | Claudin-8-like |
| itprid1 | ITPR interacting domain containing 1 |
| LOC100706611 | Leucine-rich repeat and immunoglobulin-like domain-containing nogo receptor-interacting Protein 3 |
| LOC112847085 | Uncharacterized LOC112847085 |
| LOC100696738 | DNA damage-inducible transcript 4-like protein |
| LOC102077240 | Uncharacterized LOC102077240 |
| LOC109201566 | Uncharacterized LOC109201566 |
| LOC100690816 | Fish-egg lectin |
| LOC102077915 | SLAM family member 7 |
| mfsd4a | Major facilitator superfamily domain containing 4A |
| LOC109194368 | Rho GDP-dissociation inhibitor 1 |
| LOC100701156 | Protein phosphatase 1 regulatory subunit 12B |
| LOC102079245 | Gap junction Cx32.7 protein |
| LOC100690221 | Germ cell-specific gene 1-like protein |
| LOC109197005 | Uncharacterized LOC109197005 |
| LOC102078187 | Adhesion G-protein coupled receptor G2 |
| slc41a1 | Solute carrier family 41 member 1 |
| LOC102082170 | Uncharacterized LOC102082170 |
| LOC112847623 | Small nucleolar RNA SNORD97 |
