# Supplementary material for: Anaerobic bacteria Cetobacterium sp. nov C33 plays a crucial role in the intestinal microbial balance and regulation of gene expression to immune and metabolic responses in Nile tilapia
Source: PLoS One. 2026 May 7;21(5):e0344851. doi: 10.1371/journal.pone.0344851 (PMC13152124; doi:10.1371/journal.pone.0344851)
Supplement: S2 Table — Differentially down expressed genes from Oreochromis niloticus following Cetobacterium sp. nov C33 Diet (p < 0.05), in kidney samples of tilapia fingerlings from the group fed with the Cetobacterium diet (C33D): basal diet with 1 x 10^8^ CFU/g of Cetobacterium sp. nov C33, compared to the control group fed with Control Diet (CD): basal diet; after ceasing consumption of Cetobacterium Diet for 5 days. (DOCX) [file pone.0344851.s002.docx]

S2 Table. Differentially down expressed genes.

Differentially down expressed genes from *Oreochromis niloticus* following *Cetobacterium* sp. nov C33 Diet (*p*<0.05), in kidney samples of tilapia fingerlings from the group fed with the *Cetobacterium* diet (C33D): basal diet with 1 x 10^8^ CFU/g of *Cetobacterium* sp. nov C33, compared to the control group fed with Control Diet (CD): basal diet; after ceasing consumption of *Cetobacterium* Diet for 5 days.

| Symbol | Description |
| --- | --- |
| LOC100699499 | Trypsin |
| LOC100695248 | Elastase-1 |
| LOC100694176 | Chymotrypsin-like elastase family member 2A |
| LOC102082561 | Retrovirus-related Pol polyprotein from transposon 412 |
| LOC100696175 | Chymotrypsin-like elastase family member 2A |
| LOC100708221 | Chymotrypsinogen A |
| LOC100697052 | GTP cyclohydrolase 1 |
| LOC100694894 | Maltase-glucoamylase, intestinal |
| erp27 | Endoplasmic reticulum protein 27 |
| LOC100711148 | Elastase-1 |
| LOC100698367 | Uncharacterized LOC100698367 |
| LOC102077553 | Uncharacterized LOC102077553 |
| LOC109200287 | Galactose-3-O-sulfotransferase 2-like |
| LOC109204265 | Trypsin-1 |
| LOC109199076 | Bile salt-activated lipase |
| LOC100691894 | Serine hydroxymethyltransferase, mitocondrial |
| LOC100709289 | Up-regulator of cell proliferation-like |
| LOC100711807 | Elastase-1 |
| LOC109195662 | Pancreatic alpha-amylase |
| LOC100703444 | Substance-P receptor (LOC100703444), transcript variant X1 |
| LOC100706496 | CD209 antigen-like protein E |
| LOC100701717 | Vitelline membrane outer layer protein 1 homolog |
| LOC109195030 | Prestalk protein-like |
| ebp | Cholestenol Delta-isomerase |
| msmo1 | Methylsterol monooxygenase |
| apom | Apolipoprotein M |
| LOC100698630 | Scavenger receptor cysteine-rich type 1 protein M130 |
| LOC100709771 | Insulin-like growth factor-binding protein 2-B |
| LOC100695604 | Uncharacterized LOC100695604 |
| LOC102081651 | Protein ANTAGONIST OF LIKE HETEROCHROMATIN PROTEIN 1 |
| tat | [Tyrosine aminotransferase](https://www.ncbi.nlm.nih.gov/gene/6898) |
| LOC109194556 | Uncharacterized LOC109194556 |
| LOC100697646 | 3-hydroxy-3-methylglutaryl-coenzyme A reductase |
| LOC102082976 | Uncharacterized LOC102082976 |
| LOC100711490 | Apolipoprotein A-IV |
| LOC109203547 | Cytokine-dependent hematopoietic cell linker |
| igfals | Insulin-like growth factor binding protein, acid labile subunit |
| LOC112842399 | Uncharacterized LOC112842399 |
| LOC102076100 | Plasminogen |
| serping1 | Serpin family G member 1 |
| LOC100701222 | NXPE family member 3 |
| LOC109196403 | Lactose-binding lectin l-2 |
| LOC100711310 | Basement membrane-specific heparan sulfate proteoglycan core protein |
| LOC100706658 | Alpha-2-macroglobulin |
| ctrl | Chymotrypsin-like protease CTRL-1 |
| LOC100708331 | Probable phosphatase phospho1 |
| cideb | Lipid transferase CIDEB-like |
| LOC102080308 | Uncharacterized LOC102080308 |
| mst1 | Macrophage stimulating 1 |
| LOC102076387 | Fibrinogen alpha chain |
| LOC100698164 | NECAP endocytosis associated 1 |
| LOC106097915 | Serotransferrin |
| LOC100701799 | Cytochrome P450 2J2 |
| LOC100698944 | NLR family CARD domain-containing protein 3 |
| ptgdsl1 | Lipocalin |
| LOC102078921 | Interferon-induced very large GTPase 1-like |
| ttpa | Tocopherol (alpha) transfer protein |
| LOC106097386 | Alpha-2-macroglobulin |
| LOC102082008 | Poly(U)-specific endoribonuclease |
| LOC112846466 | Uncharacterized LOC112846466 |
| LOC100701614 | Granzyme B |
| LOC100706258 | Galactose-specific lectin nattectin |
| onecut2 | One cut homeobox 2 |
| LOC102080294 | Uncharacterized LOC102080294 |
| LOC100708498 | Olfactory receptor 52K1-lik |
| LOC100694785 | Endonuclease domain-containing 1 protein |
| LOC100712280 | 6-phosphofructo-2-kinase/fructose-2,6-bisphosphatase |
| LOC100696180 | Fibronectin |
| LOC112844704 | Uncharacterized LOC112844704 |
| LOC100706576 | Indoleamine 2,3-dioxygenase 2 |
| rbp4 | Polyadenylate-binding protein RBP47B |
| LOC100708926 | Uncharacterized LOC100708926 |
| LOC112845845 | 18S ribosomal RNA |
| gcgr | Glucagon receptor a |
| LOC100704211 | Transmembrane 6 superfamily member |
| LOC100700077 | Fatty acid desaturase 2 |
| LOC109204450 | Uncharacterized LOC109204450 |
| LOC100708134 | CMP-N-acetylneuraminate-beta-galactosamide-alpha-2,3-sialyltransferase 1-like |
| LOC106096818 | Apoptosis-associated speck-like protein containing a CARD |
| LOC100712028 | Apolipoprotein C-I |
| LOC100699490 | Somatostatin receptor type 2 |
| adamts13 | ADAM metallopeptidase with thrombospondin type 1 motif 13 |
| LOC112843666 | 18S ribosomal RNA |
| LOC102082239 | Uncharacterized LOC102082239 |
| LOC100697673 | Uncharacterized LOC100697673 |
| LOC106097433 | Uncharacterized LOC106097433 |
| LOC109204091 | Macrophage mannose receptor 1-like |
| lipc | Lipase C, hepatic type |
| LOC102076307 | Uncharacterized LOC102076307 |
| LOC100708634 | Apolipoprotein A-I |
| LOC100694687 | Cytochrome P450 2F2 |
| LOC100699875 | Perforin-1 |
| LOC109199406 | Uncharacterized LOC109199406 |
| LOC112848153 | Uncharacterized LOC112848153 |
| LOC102076719 | Barrier-to-autointegration factor-like protein |
| serpina10 | Serpin family A member 10 |
| LOC102081389 | Uncharacterized LOC102081389 |
| LOC102079326 | Alpha-2-macroglobulin |
| LOC100701144 | Leucine-rich alpha-2-glycoprotein |
| LOC100712102 | Serine protease inhibitor A3K |
| LOC100692295 | Selenoprotein Pb |
| LOC109198891 | Uncharacterized LOC109198891 |
| LOC106096990 | Uncharacterized LOC106096990 |
| LOC112845859 | 18S ribosomal RNA |
| gal3st1 | Galactose-3-O-sulfotransferase 1 |
| spp2 | [Secreted phosphoprotein 2](https://www.ncbi.nlm.nih.gov/gene/6694) |
| LOC100695163 | Uncharacterized LOC100695163 |
| LOC100702313 | Prothrombin |
| LOC106099041 | Interferon-induced very large GTPase 1 |
| abcg8 | ATP-binding cassette, sub-family G (WHITE), member 8 |
| spock2 | SPARC (osteonectin), cwcv and kazal like domains proteoglycan 2 |
| LOC100690101 | Type-4 ice-structuring protein |
| LOC102076087 | Alpha-2-macroglobulin |
| LOC100702684 | Ceruloplasmin |
| LOC100705436 | Betaine--homocysteine S-methyltransferase 1 |
| LOC100699903 | Carboxypeptidase B2 |
| ccdc172 | Coiled-coil domain containing 172 |
| LOC112845702 | 28S ribosomal RNA |
| LOC100712040 | Acidic amino acid decarboxylase GADL1 |
| LOC100701251 | Alpha-2-macroglobulin |
| LOC100701889 | Up-regulator of cell proliferation-like |
| LOC100698693 | Major histocompatibility complex class I-related gene protein |
| LOC100697261 | Acetyl-coenzyme A synthetase, cytoplasmic |
| LOC100704493 | Dimethylaniline monooxygenase [N-oxide-forming] 5 |
| LOC100695437 | Golgin subfamily A member 4 |
| LOC102077690 | Up-regulator of cell proliferation-like |
| LOC100702432 | Hemopexin |
| qsox1 | Quiescin sulfhydryl oxidase 1 |
| LOC100704850 | Glutaminase kidney isoform, mitochondrial |
| LOC100702671 | Plasminogen |
| pah | Phenylalanine hydroxylase |
| cpb1 | Carboxypeptidase B1 (tissue) |
| LOC109198671 | Uncharacterized LOC109198671 |
| iffo1 | Intermediate filament family orphan 1 |
| LOC112843665 | 18S ribosomal RNA |
| rbp2 | Retinol binding protein |
| LOC100712382 | Coagulation factor XI |
| LOC100694471 | Uncharacterized LOC100694471 |
| apo14kda | Apolipoprotein A-II |
| f5 | Coagulation factor V |
| LOC106096476 | Coagulation factor X |
| LOC102079469 | Serine protease inhibitor Kazal-type 2 |
| LOC100692275 | Fucolectin-4 |
| LOC112843391 | Uncharacterized LOC112843391 |
| LOC100705158 | Microfibril-associated glycoprotein 4 |
| LOC100691917 | Complement C3 |
| cdo1 | Cysteine dioxygenase type 1 |
| LOC106096858 | Prothrombin |
| ppp1r3g | Protein phosphatase 1 regulatory subunit 3G |
| LOC100699900 | Zona pellucida sperm-binding protein 3 |
| LOC109202857 | Plancitoxin-1-like |
| fadsd6 | Delta-6 fatty acyl desaturase |
| LOC100709003 | Complement C3 |
| LOC112845127 | 18S ribosomal RNA |
| LOC100701948 | GTP cyclohydrolase 1 |
| LOC102078215 | Trichohyalin-like |
| LOC109203929 | Uncharacterized LOC109203929 |
| LOC100703674 | Cytochrome P450 3A40 |
| LOC100706759 | Complement C1r subcomponent |
| LOC106097902 | Alpha-1-antitrypsin homolog |
| LOC100691300 | Vitellogenin |
| LOC112843831 | Uncharacterized LOC112843831 |
| LOC100691737 | Perlucin-like protein |
| LOC102076085 | Uncharacterized LOC102076085 |
| LOC100694361 | SEC14-like protein 2 |
| LOC106097655 | Protein C2-DOMAIN ABA-RELATED 3 |
| etnppl | Ethanolamine-phosphate phospho-lyase |
| LOC100705815 | Kininogen-1 |
| LOC100703116 | Vitamin K-dependent protein Z |
| LOC100710669 | Coagulation factor IX |
| LOC112848257 | Complement C1r subcomponent-like |
| LOC100694381 | Coagulation factor IX |
| c8a | Complement C8 alpha chain |
| shbg | Sex hormone-binding globulin |
| LOC100691584 | Chymotrypsinogen A |
| LOC100707011 | Solute carrier organic anion transporter family member 1C1 |
| LOC100699343 | Beta-2-glycoprotein 1 |
| LOC100710661 | 3-oxo-5-beta-steroid 4-dehydrogenase |
| LOC109197780 | Uncharacterized LOC109197780 |
| LOC100697522 | N-acetylmuramoyl-L-alanine amidase |
| LOC109195166 | Complement factor H |
| LOC100701236 | Multidrug resistance-associated protein 9 |
| LOC106098940 | Protein C2-DOMAIN ABA-RELATED 3-like |
| LOC100703849 | Carcinoembryonic antigen-related cell adhesion molecule 5-like |
| angptl3 | Angiopoietin-like 3 |
| LOC109194488 | Uncharacterized LOC109194488 |
| LOC112842563 | 18S ribosomal RNA |
| fasn | Fatty acid synthase |
| LOC100708305 | UDP-glucuronosyltransferase 2C1 |
| LOC106098933 | Inter-alpha-trypsin inhibitor heavy chain H3 |
| LOC100710427 | Complement factor H-related protein 5 |
| tbtbp | Putative TBT binding protein |
| LOC100702232 | Carboxypeptidase N subunit 2 |
| LOC100697799 | High choriolytic enzyme 1 |
| LOC100698642 | Multiple inositol polyphosphate phosphatase 1 |
| LOC100706310 | Complement factor H |
| LOC109203946 | Zinc finger BED domain-containing protein 1-like |
| LOC100699793 | Trypsin-2 |
| c8b | Complement component 8 subunit beta |
| LOC100692186 | Complement C3 |
| apoc2 | Apolipoprotein C-II |
| LOC112841994 | Uncharacterized LOC112841994 |
| LOC100710487 | Sodium- and chloride-dependent GABA transporter 2 |
| LOC112842620 | Uncharacterized LOC112842620 |
| LOC100690088 | Alpha-2-macroglobulin-like |
| LOC112841650 | Myelin-oligodendrocyte glycoprotein-like |
| LOC100691268 | Uncharacterized LOC100691268 |
| qprt | Quinolinate phosphoribosyltransferase |
| LOC112841635 | Inter-alpha-trypsin inhibitor heavy chain H3-like |
| LOC100707322 | Complement factor H-related protein 1 |
| LOC100708633 | Carboxypeptidase A1 |
| LOC100705080 | Myomesin-3 |
| LOC100698407 | NLR family CARD domain-containing protein 3 |
| LOC100695795 | Somatostatin receptor type 2 |
| LOC102080172 | Uncharacterized LOC102080172 |
| tmprss6 | Transmembrane serine protease 6 |
| LOC102080707 | Deleted in malignant brain tumors 1 protein |
| LOC100695916 | Chymotrypsin-like elastase family member 2A |
| LOC102077759 | Uncharacterized LOC102077759 |
| LOC102079839 | Inter-alpha-trypsin inhibitor heavy chain H3 |
| LOC100703020 | Nucleoside diphosphate kinase, mitochondrial |
| LOC109204569 | UDP-glucuronosyltransferase 2B31-like |
| LOC100690724 | Hepatocyte nuclear factor 4-beta |
| LOC100703931 | Coagulation factor VII |
| comtd1 | Catechol-O-methyltransferase domain containing 1 |
| LOC100703919 | Complement C4 |
| LOC100710422 | Inhibin beta B chain |
| LOC100703742 | Selenoprotein M |
| LOC112845802 | 18S ribosomal RNA |
| LOC100691312 | Agouti-related protein |
| LOC100690147 | Lysozyme C |
| LOC100697699 | Complement C2 |
| proc | Protein C, inactivator of coagulation factors Va and VIIIa |
| LOC102081243 | Alpha-2-macroglobulin |
| LOC109202378 | E3 ubiquitin/ISG15 ligase TRIM25 |
| LOC100708276 | Mid1-interacting protein 1-B |
| LOC102082601 | Group 3 secretory phospholipase A2 |
| LOC109194451 | Fetuin-B |
| LOC100695729 | Acyl-CoA desaturase |
| LOC100702388 | Complement C1q-like protein 3 |
| LOC100706937 | Leukocyte cell-derived chemotaxin-2 |
| LOC109195663 | Pancreatic alpha-amylase-like |
| gys2 | Glycogen synthase 2 |
| LOC102082816 | Up-regulator of cell proliferation-like |
| LOC109194289 | Pancreatic alpha-amylase |
| LOC112845901 | 18S ribosomal RNA |
| LOC112843769 | Coiled-coil domain-containing protein 106-like |
| LOC106098411 | Interferon-induced very large GTPase 1-like |
| slc22a16 | Solute carrier family 22 member 16 |
| cfi | Complement factor I |
| LOC100691786 | Bile salt export pump |
| LOC100697028 | Alpha-2-antiplasmin |
| LOC109194549 | Uncharacterized LOC109194549 |
| LOC102077119 | Up-regulator of cell proliferation-like |
| LOC106097916 | Deleted in malignant brain tumors 1 protein |
| LOC100534516 | Serine protease 1 |
| LOC100703411 | Bile salt-activated lipase |
| LOC112847025 | Uncharacterized LOC112847025 |
| LOC106097376 | Uncharacterized LOC106097376 |
| LOC100701822 | C-type lectin domain family 10 member A |
| LOC102078829 | High choriolytic enzyme 1 |
| LOC100703791 | Dual specificity phosphatase DUPD1 |
| LOC112848225 | Uncharacterized LOC112848225 |
| cldn1 | Claudin 12 |
| LOC100701014 | Pancreatic alpha-amylase |
| LOC109195164 | NXPE family member 3 |
| LOC100705483 | N-acetylmuramoyl-L-alanine amidase |
| LOC109199077 | Bile salt-activated lipase-like |
| LOC100708966 | Uncharacterized LOC100708966 |
| LOC100706232 | CD209 antigen |
| LOC100708379 | Fetuin-B |
| LOC112847572 | Uncharacterized LOC112847572 |
| LOC100690942 | Beta-microseminoprotein |
| LOC100703770 | Acidic mammalian chitinase |
| LOC112845817 | 18S ribosomal RNA |
| LOC100534520 | Trypsin-2 |
| LOC112842746 | Esmocollin-2-like |
