# Supplementary material for: Anaerobic bacteria Cetobacterium sp. nov C33 plays a crucial role in the intestinal microbial balance and regulation of gene expression to immune and metabolic responses in Nile tilapia
Source: PLoS One. 2026 May 7;21(5):e0344851. doi: 10.1371/journal.pone.0344851 (PMC13152124; doi:10.1371/journal.pone.0344851)
Supplement: Attachment — Submitted filename: Response letter.pdf [file pone.0344851.s012.pdf]

August 12, 2025

Dr. Amel Mohamed El Asely

Academic Editor

PLOS ONE

Dear Dr. El Asely,

I appreciate your consideration of our manuscript for publication. In attention to reviewers’ comments about our research manuscript PONE-D-25-18938, entitled "The anaerobic bacterium *Cetobacterium* sp. nov C33 plays a crucial role in intestinal microbial balance and the regulation of gene expression for immune and metabolic responses in Nile tilapia," by Mario Andrés Colorado Gómez, Javier Fernando Melo-Bolívar, Ruth Yolanda Ruíz Pardo, Howard Junca, Juan F. Alzate, and Luisa Villamil. We respond, their comments.

We respectfully request an additional week to upload the sequence files to SRA NCBI. However, the data will be available in our OneDrive folder if reviewers request it.

King regards,

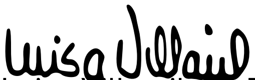

Luisa Villamil, Ph.D.
School of Engineering
Universidad de La Sabana, Colombia, South America

Journal Requirements:

Comment 1. Please ensure that your manuscript meets PLOS ONE's style requirements, including those for file naming.

Response 1. We reviewed the entire manuscript to ensure it complies with PLOS ONE style requirements, including file naming requirements.

Comment 2. To comply with PLOS ONE submissions requirements, in your Methods section, please provide additional information regarding the experiments involving animals and ensure you have included details on (1) methods of sacrifice, and (2) efforts to alleviate suffering.

Response 2. We include in lines 113-126 the details of the methods of sacrifice and the efforts to alleviate suffering, this is the paragraph: "The experiments involved animals, therefore, the methods and protocols were carried out according to ARRIVE guidelines and regulations [22] and all methods were performed in accordance with the relevant guidelines and regulations of the Management and Use of Laboratory Animals of Colombia and complied with Colombia's existing laws and regulations for biological research. Furthermore, the euthanasia protocol sought to reduce fish pain in the experiment, implementing euthanasia as an animal welfare method. Therefore, an overdose of tricaine methanesulfonate (MS-222, 200 mg L-1) [9] was administered by prolonged immersion. Fish were submerged in a tricaine solution for more than 5 minutes or until no opercular movement was observed. Likewise, for the efforts to alleviate suffering, two of the 3R criteria were taken into account: reduction, which refers to using the smallest possible number of animals to obtain a meaningful and valid result [23]. Refinement, which is related to improving handling techniques and reducing pain and suffering in research animals, was also considered. Welfare criteria were established for the fish, such as morphological characteristics (loss of scales, body lesions, etc.) and behavioral characteristics that could be caused by the experimental handling of the animals, which are under stress in certain situations".

Comment 3. Thank you for stating the following financial disclosure: [This research was funded by the Sistema Nacional de Regalias (SGR): Ministerio de Hacienda de Colombia y Ministerio de Ciencias y Tecnología e Innovación de Colombia. Project code before the National Bank of Investment Programs and Projects BPIN: 2020000100487. The project was carried out by the Universidad de La Sabana and the Servicio Nacional de Aprendizaje -SENA- Regional La Guajira.].

Please state what role the funders took in the study. If the funders had no role, please state: "The funders had no role in study design, data collection and analysis, decision to publish, or preparation of the manuscript."

Response 3. We wrote in the line 792-793: “The funders had no role in study design, data collection and analysis, decision to publish, or preparation of the manuscript”.

Comment 4. In the online submission form, you indicated that [Data will be made available on request.].

All PLOS journals now require all data underlying the findings described in their manuscript to be freely available to other researchers, either 1. In a public repository, 2. Within the manuscript itself, or 3. Uploaded as supplementary information.

Response 4: We Uploaded all data as supplementary information.

Metataxonomic data:

Files: ASV_Ceto, Meta_Ceto, Taxa_Ceto, Data Metataxonomy, Summary_file

Transcriptomics data:

Files: Data, Desing, Metadata RNAseq

Comment 5. When completing the data availability statement of the submission form, you indicated that you will make your data available on acceptance. We strongly recommend all authors decide on a data sharing plan before acceptance, as the process can be lengthy and hold up publication timelines. Please note that, though access restrictions are acceptable now, your entire data will need to be made freely accessible if your manuscript is accepted for publication. This policy applies to all data except where public deposition would breach compliance with the protocol approved by your research ethics board. If you are unable to adhere to our open data policy, please kindly revise your statement to explain your reasoning and we will seek the editor's input on an exemption. Please be assured that, once you have provided your new statement, the assessment of your exemption will not hold up the peer review process.

Response 5: We respectfully request an additional week to upload the sequence files to SRA NCBI. However, the data will be available in our OneDrive folder if reviewers request it. Please request access to the OneDrive repository by emailing luisa.villamil@unisabana.edu.co

Metataxonomic data:

Sample	Treatment	File	File	Sample ID
C7	Control	C7_1.fq.gz	C7_2.fq.gz	MCD1
C8	Control	C8_1.fq.gz	C8_2.fq.gz	MCD2
C9	Control	C9_1.fq.gz	C9_2.fq.gz	MCD3
D2	*Cetobacterium*	D2_1.fq.gz	D2_2.fq.gz	MC33D1
D3	*Cetobacterium*	D3_1.fq.gz	D3_2.fq.gz	MC33D2

D4	*Cetobacterium*	D4_1.fq.gz	D4_2.fq.gz	MC33D3
D5	*Cetobacterium*	D5_1.fq.gz	D5_2.fq.gz	MC33D4

Transcriptomic data:

Sample	Treatment	File	File	Sample ID
R42	Control	R42_1.fq.gz	R42_2.fq.gz	TCD1
R47	Control	R47_1.fq.gz	R47_2.fq.gz	TCD2
R53	Control	R53_1.fq.gz	R53_2.fq.gz	TCD3
R57	*Cetobacterium*	R57_1.fq.gz	R57_2.fq.gz	TC33D1
R58	*Cetobacterium*	R58_1.fq.gz	R58_2.fq.gz	TC33D2
R59	*Cetobacterium*	R59_1.fq.gz	R59_2.fq.gz	TC33D3
R60	*Cetobacterium*	R60_1.fq.gz	R60_2.fq.gz	TC33D4

Response to Reviewer

Thank you very much for taking the time to review this manuscript. Please find the detailed responses below and the corresponding revisions and changes in the re-submitted files.

Response to Reviewer 1 Comments

Comments 1: Even if the current study offers an effective probiotic to improve fish development, immunity, and reproduction, it has numerous limitations that should be taken into account, including the following implications

1- The probiotic effects of *Cetobacterium* sp. nov C33 were evaluated over a short supplementation period, which limits understanding of its long-term safety, stability, and sustained efficacy in tilapia.
   Response: Short-term administration of microorganisms can also offer advantages in terms of investment in treatment, especially when positive results are found. However, the study of long-term administration will also be very interesting to analyze in the longer term.
2- The study was conducted exclusively on fingerlings, so the applicability of findings to juveniles or adults remains uncertain.
   Response: The research focuses on fingerlings due to their susceptibility to disease. Furthermore, differences in the microbiota at different culture stages have not been studied, so evaluating the effect of *Cetobacterium* sp. nov C33 at other culture stages and on a larger scale is very interesting and could be done in future studies.
3- The trial was conducted under controlled laboratory or hatchery settings, which may not fully capture the complexity and environmental variability of commercial aquaculture systems.
   Response: Agreed, most probiotic studies are conducted on a laboratory scale rather than in production settings. This is related to ensuring the safety of the microorganisms being evaluated, especially *Cetobacterium* sp. nov C33, which has been little studied. Furthermore, scaling up production of *Cetobacterium* sp. nov C33 for commercial-scale testing requires much more research, but given the positive results, we want to move forward in this direction.
4- Although immune-related gene expression was assessed, the study did not include a disease challenge to directly measure protection against specific pathogens or real-world disease resistance.
   Response: At the time of the research, there was no permission from the institutional ethics committee to conduct the experimental challenge.

5- While 16S rRNA sequencing provided taxonomic shifts in microbiota composition, it did not include metagenomic or metabolomic analyses to clarify functional changes in microbial activity or interactions.
   Response:
6- Findings are specific to Nile tilapia, and their generalizability to other aquaculture species is unknown without further comparative studies.
   Response: Our findings are specific to Nile tilapia and we do not generalize them to other species.
7- So, it is highly recommended to have a paragraph to discuss the major limitations of the present study before the section of the Conclusion.
   Response: We include the study's limitations in a paragraph in Line 749-762. We appreciate the peer reviewers' observations, as they highlight new opportunities to continue our research.

Minor comments:

1- English proofreading is highly recommended.

Response: The authors have revised the manuscript according to the reviewers' comments, and the quality is improved. However, if the editor deems it appropriate, after the review we would hire a writing revision service as recommended by the journal.

2- No statistical analysis, so please analyze that data.

Response: Statistical analysis was carried out in included in the submitted manuscript, nevertheless the methodology was improved to described the analysys carried out. Also, we included in the Tables 1 and 2 the Statistical data for differential significantly for genera taxon, Furthermore, in supplementary tables S1 and S2, we included the Statical data for differential significantly for genes upregulated and downregulated.

3- No line numbers.

Response: We applied the Line numbers according to Plos One's instructions.

4- Please prepare the manuscript according to Plos One's instructions. For example the title page does not follow the guidelines.

Response: We applicated the guidelines of Plos One´s instructions

5- Check the grammer the 2nd paragraph in the introduction “The intestinal bacterial composition in tilapia is has been previously described”.

Response: We correct grammar and wrote in Line 57-59: “Previous research has indicated that the intestinal bacterial composition of tilapia is primarily composed of three phyla: Fusobacteria, Bacteroidota, and Proteobacteria”.

6- Please add brackets for SVCV to indicate that this is abbreviation for spring viraemia of carp virus (3rd paragraph in the introduction)

Response: We added brackets for SVCV in Line 70.

7- Check the grammer the 2nd paragraph in the introduction “y Though the search or new potential probiotics” I believe you mean “through the search for new potential probiotics”, so please correct it.

Response: We wrote “through the search for new potential probiotics” in the Line 97-98.

8- The references you used to plan your experiments are missing from the entire methods section; please update and add them across the entire manuscript.

Response: We included the references in the methods section. The references are:

9. Melo-Bolívar, J. F., Ruiz Pardo, R. Y., Quintanilla-Carvajal, M. X., Díaz, L. E., Alzate, J. F., Junca, H., Rodríguez Orjuela, J. A., & Villamil Diaz, L. M. (2023). Evaluation of dietary single probiotic isolates and probiotic multistrain consortia in growth performance, gut histology, gut microbiota, immune regulation, and infection resistance of Nile tilapia, Oreochromis niloticus, shows superior monostrain performance. Fish & Shellfish Immunology, 140, 108928. https://doi.org/10.1016/J.FSI.2023.108928

19. FA, R. (2018). Genome-Nutrifortified Diets-Their Disease Protection and Remedy Potential. Journal of Probiotics & Health, 06(02). https://doi.org/10.4172/2329-8901.1000204

22. Kilkenny, C., Browne, W. J., Cuthill, I. C., Emerson, M., & Altman, D. G. Improving bioscience research reporting: the ARRIVE guidelines for reporting animal research. PLoS biology. (2010; 8(6), e1000412. https://doi.org/10.1371/journal.pbio.1000412

23. Rose, M., Everitt, J., Hedrich, H., Schofield, J., Dennis, M., Scott, E., & Griffin, G. (2013). ICLAS working group on harmonization: International guidance concerning the production care and use of genetically-altered animals. Laboratory Animals, 47(3), 146–152. https://doi.org/10.1177/0023677213479338

25. Sharifuzzaman, S. M., & Austin, B. (2010). Development of protection in rainbow trout (Oncorhynchus mykiss, Walbaum) to Vibrio anguillarum following use of the probiotic Kocuria SM1. Fish & Shellfish Immunology, 29(2), 212–216. https://doi.org/10.1016/J.FSI.2010.03.008

31. Zhou, R., Ng, S. K., Sung, J. J. Y., Goh, W. W. bin, & Wong, S. H. (2023). Data pre-processing for analyzing microbiome data – A mini review. Computational and

Structural Biotechnology Journal, 21, 4804–4815. https://doi.org/10.1016/J.CSBJ.2023.10.001

32. Cao, Q. X., Sun, X., Rajesh, K., Chalasani, N., Gelow, K., Katz, B., Shah, V. H., Sanyal, A. J., & Smirnova, E. (2020). Effects of microbiome rare taxa filtering on statistical analysis. https://doi.org/10.21203/RS.3.RS-34781/V1

33. Cameron, E. S., Schmidt, P. J., Tremblay, B. J. M., Emelko, M. B., & Müller, K. M. (2021). Enhancing diversity analysis by repeatedly rarefying next generation sequencing data describing microbial communities. Scientific Reports, 11(1). https://doi.org/10.1038/S41598-021-01636-1

34. Cappellato, M., Baruzzo, G., & Camillo, B. di. (2022). Investigating differential abundance methods in microbiome data: A benchmark study. PLoS Computational Biology, 18(9), e1010467. https://doi.org/10.1371/JOURNAL.PCBI.1010467

9- Keep the conclusion to a minimum to make it more representative and focused

Response: We reduce the conclusion chapter. Lines: 764-774.

Response to Reviewer 2 Comments

Comment 1: The Paper entitled Anaerobic bacteria Cetobacterium sp. nov C33 plays a crucial role in the intestinal microbial balance and regulation of gene expression to immune and metabolic responses in Nile Tilapia which has been submitted to publish in PLOS One presents a good investigative efforts but the hypothesis derived from the obtained data is scientifically and logically wrong very likely because there is a gap in present understanding on knowledge.

Response 1: We, according to the scope of our project, did not present a hypothesis, we reference the possibility that *Cetobacterium* could be a candidate to be considered as a probiotic for use in fish. We removed the word hypothesis from the document to avoid confusion for the reader. However, according to the response obtained at the transcriptomic level, we express the possibility that a greater abundance of *Cetobacterium* isolated from specimens of the same species generates a positive effect on the health of fish.

Comment 2: Low cell population e.g.10^8cells/g of Anaerobic bacteria *Cetobacterium* sp. nov C33 present in feed are practically too less to enable the fed innoculum to appear as dominating species in Nile Tilapia particularly in the case when it requires anaerobic condition to grow at 28°C whereas the temperature of fish body is usually lower than 28°C with limited availability of oxygen.

Response 2: Previous studies conducted within our research group, utilizing culture-independent techniques, revealed that *Cetobacterium* was a predominant genus within the intestinal microbiota, Melo-Bolivar *et al*. (2019) reported that "The most abundant bacterium in the first days of the CFCEC, was Cetobacterium sp.". On the other hand, for use the probiotics in Nile tilapia, various authors used bacteria in different quantities, such as Wang *et al*. (2008) who use *Enterococcus faecium* 1 × 10^{7} UFC/mL during 40 day and achieve final weight increase. Hassaan *et al*. (2018) used *Bacillus subtilis* 1,1 × 10^{5} UFC/g for 84 day and reach improvement of growth performance. Van Doan *et al*. (2019) used *Lactobacillus plantarum* 1 × 10^{8} UFC/g during 84 days and reach improvement of growth performance. Finally, Sewaka *et al*. (2019) used *Lactobacillus rhamnosus* 1 × 10^{8} UFC/g during 2 weeks, and enhancement of histo-morphological intestinal parameters.

References

Hassaan, M.S.; Soltan, M.A.; Jarmołowicz, S.; Abdo, H.S. Combined effects of dietary malic acid and *Bacillus subtilis* on growth, gut microbiota and blood parameters of Nile tilapia (*Oreochromis niloticus*). Aquac. Nutr (2018) *24*, 83–93

Melo-Bolívar, J. F., Ruiz Pardo, R. Y., Hume, M. E., Nisbet, D. J., Rodríguez-Villamizar, F., Alzate, J. F., Junca, H., & Villamil Díaz, L. M. Establishment and characterization of a competitive exclusion bacterial culture derived from Nile tilapia (*Oreochromis niloticus*) gut microbiomes showing antibacterial activity against pathogenic *Streptococcus agalactiae*. PLOS ONE. (2019). 14(5), e0215375.

Sewaka, M.; Trullas, C.; Chotiko, A.; Rodkhum, C.; Chansue, N.; Boonanuntanasam, S.; Pirarat, N. Efficacy of synbiotic *Jerusalem artichoke* and *Lactobacillus rhamnosus GG*-supplemented diets on growth performance, serum biochemical parameters, intestinal morphology, immune parameters and protection against *Aeromonas veronii* in juvenile red tilapia (*Oreochromis spp*.). Fish Shellfish Immunol (2019). *86*, 260–268

Van Doan, H.; Kurian, A.; Hoseinifar, S.H.; Sel-audom, M.; Jaturasitha, S.; Tongsiri, S.; Ringø, E. Dietary inclusion of orange peels derived pectin and *Lactobacillus plantarum* for Nile tilapia (*Oreochromis niloticus*) cultured under indoor biofloc systems. Aquaculture (2019). *508*, 98–105.

Wang, Y.-B.; Tian, Z.-Q.; Yao, J.-T.; Li, W. Effect of probiotics, Enteroccus faecium, on tilapia (Oreochromis niloticus) growth performance and immune response. Aquaculture (2008). 277, 203–207

Comment 3: However these cells by virtue of their high hydrophobicity can make clusters having potential to adhere with cells membrane of cells lining the gut effecting Oxygen, cholesterol and different metallic ions transport particularly Copper and Iron across the cell membranes effecting the host cells functions of target cells and also interconnected cells translating the impact across the organs connected through body systems modulating genes network operations by virtue of copper and iron balance in associated and dissociated forms effected differently by oxygen availability at cellular level, enabling cells to attain new phenotypes with altered homeostasis supported environment leading to the shift in microbial populations and shift in body cell functions over the period attaining altered normal. In response of stress conditions in gut the anaerobic bacteria Cetobacterium sp. nov C33 produce pigments and other secondary metabolites e.g. chelating agents enzymes etc which alters the iron availability and its accessibility in the cell environment which is is evident by up regulation and down regulation of different genes without activating an inflammatory pathway inspite of involving immune system of the host and without uncontrolled fluctuation in energy demand in body cells subsets. Using yeast as a model, it has already been established that CTR1,(CTR1 gene encodes high affinity copper transporter) SOD1, superoxide dismutase 1 gene, GSH encoding genes and cholesterol cellular biosynthesis genes which are interdependently co-regulated at transcriptional level to sustain metallic ions homeostasis, energy homeostasis, cell s aging leading to cell evolution or cell

death. Papers revealing the underlying molecular mechanisms have already been submitted.

Response 3: *Cetobacterium* sp. nov C33 does not produce pigments and no regulation with metal ions was found, neither in the literature nor through genome review. We applied antiSMASH, a bioinformatics tool that allows the detection of gene clusters associated with the biosynthesis of secondary metabolites, including siderophores. It is used to analyze complete or assembled bacterial genomes and search for genes encoding enzymes involved in siderophore synthesis. *Cetobacterium* sp. nov C33, we did not find siderophores, but the results showed four regions in the genomes like glycerol dehydrogenase. (Region 1 – NRPS, RFV38_04915 glycerol dehydrogenase Locus tag: RFV38_04915 Protein ID: MDX8335839.1 Gene: None Location: 17,505 - 18,581, (total: 1077 nt)); Region 1 - NRPS-like, Location: 67,328 - 69,838, (total: 2511 nt); Region 1 - RiPP-like Location: 32,143 - 33,093, (total: 951 nt); Region 1 - terpene-precursor. Location: 14,714 - 15,595, (total: 882 nt).

NRPS-like (Nonribosomal Peptide Synthetase-like) and siderophores are both involved in the biosynthesis of molecules with distinct functions, but they differ in their structure, biosynthesis pathways, and specific roles. NRPS-like systems produce a diverse array of bioactive peptides, including antibiotics and immunosuppressants, while siderophores are primarily involved in iron acquisition by binding and transporting ferric iron (Fe3+).

It is important to highlight that *Cetobacterium* sp nov C33 was isolated from Oreochromis niloticus (Colorado Gómez *et al*., 2023) and that *Cetobacterium* has been reported as the most abundant genus in the gut microbiota of Nile tilapia (Melo-Bolívar *et al*., 2019). *Cetobacterium* itself has not been reported as an infectious agent in fish (Xiao *et al*., 2021). I

References

Balcázar JL, Vendrell D, de Blas I, Ruiz-Zarzuela I, Gironé s O, Muzquiz JL. Immune modulation by probiotic strains: Quantification of phagocytosis of Aeromonas salmonicida by leukocytes isolated from gut of rainbow trout (Oncorhynchus mykiss) using a radiolabelling assay. Comp Immunol Microbiol Infect Dis (2006) 29(5):335–43. doi: 10.1016/j.cimid.2006.09.004

Cerezuela R, Guardiola FA, Cuesta A, Esteban MA. Enrichment of gilthead seabream (Sparus aurata L.) diet with palm fruit extract andn probiotics: effects on skin mucosal immunity. Fish Shellfish Immunol (2016) 49:100–9. doi: 10.1016/j.fsi.2015.12.028

Colorado Gómez, M. A., Melo-Bolívar, J. F., Ruíz Pardo, R. Y., Rodriguez, J. A., & Villamil, L. M. Unveiling the Probiotic Potential of the Anaerobic Bacterium

Cetobacterium sp. nov. C33 for Enhancing Nile Tilapia (Oreochromis niloticus) Cultures. Microorganisms. 2023; 11(12), 2922. https://doi.org/10.3390/MICROORGANISMS11122922/S1

Dawood MAO, Abo-Al-Ela HG, Hasan MT. Modulation of transcriptomic profile in aquatic animals: probiotics, prebiotics and symbiotic scenarios. Fish Shellfish Immunol (2020) 97:268–82. doi: 10.1016/j.fsi.2019.12.054

Di J, Chu Z, Zhang S, Huang J, Du H, Wei Q. Evaluation of the potential probiotic Bacillus subtilis isolated from two ancient sturgeons on growth performance, serum immunity and disease resistance of Acipenser dabryanus. Fish Shellfish Immunol (2019) 93:711–9. doi: 10.1016/j.fsi.2019.08.020

Gobi N, Vaseeharan B, Chen J-C, Rekha R, Vijayakumar S, Anjugam M, et al. Dietary supplementation of probiotic Bacillus licheniformis Dahb1 improves growth performance, mucus and serum immune parameters, antioxidant enzyme activity as well as resistance against Aeromonas hydrophila in tilapia Oreochromis mossambicus. Fish Shellfish Immunol (2018) 74:501–8. doi: 10.1016/j.fsi.2017.12.066

Gomez D, Sunyer JO, Salinas I. The mucosal immune system of fish: the evolution of tolerating commensals while fighting pathogens. Fish Shellfish Immunol (2013) 35(6):1729–39. doi: 10.1016/j.fsi.2013.09.032 24.

Gómez GD, Balcázar JL. A review on the interactions between gut microbiota and innate immunity of fish. FEMS Immunol Med Microbiol (2008) 52 (2):145–54. doi: 10.111/j.1574-695X.2007.00343.x

Hosseini M, Miandare HK, Hoseinifar SH, Yarahmadi P. Dietary Lactobacillus acidophilus modulated skin mucus protein profile, immune and appetite genes expression in gold fish (Carassius auratus gibelio). Fish Shellfish Immunol (2016) 59:149–54. doi: 10.1016/j.fsi.2016.10.026

Jiang Y, Zhou S, Chu W. The effects of dietary Bacillus cereus QSI-1 on skin mucus proteins profile and immune response in Crucian Carp (Carassius auratus). Fish Shellfish Immunol (2019) 89:319–25. doi: 10.1016/j.fsi. 2019.04.014

Kuebutornye FKA, Wang Z, Lu Y, Abarike ED, Sakyi MW, Li Y, et al. Effects of three host-associated Bacillus species on mucosal immunity and gut health of Nile tilapia, Oreochromis niloticus and its resistance against Aeromonas hydrophila infection. Fish Shellfish Immunol (2020) 97:83–95. doi: 10.1016/ j.fsi.2019.12.046

Kuebutornye FKA, Wang Z, Lu Y, Abarike ED, Sakyi MW, Li Y, et al. Effects of three host-associated Bacillus species on mucosal immunity and gut health of Nile tilapia, Oreochromis niloticus and its resistance against Aeromonas hydrophila infection. Fish Shellfish Immunol (2020) 97:83–95. doi: 10.1016/ j.fsi.2019.12.046

Lazado CC, Caipang CMA. Mucosal immunity and probiotics in fish. Fish Shellfish Immunol (2014) 39(1):78–89. doi: 10.1016/j.fsi.2014.04.015

Lescheid DW. Probiotics as regulators of inflammation: a review. Funct Food Health Dis (2014). 4(7):299–311. doi: 10.31989/ffhd.v4i7.2

Li J, Wu Z-B, Zhang Z, Zha J-W, Qu S-Y, Qi X-Z, et al. Effects of potential probiotic Bacillus velezensis K2 on growth, immunity and resistance to Vibrio harveyi infection of hybrid grouper (Epinephelus lanceolatus♂ × E. fuscoguttatus♀). Fish Shellfish Immunol (2019). 93:1047–55. doi: 10.1016/j.fsi.2019.08.047

Melo-Bolívar, J. F., Ruiz Pardo, R. Y., Hume, M. E., Nisbet, D. J., Rodríguez-Villamizar, F., Alzate, J. F., Junca, H., & Villamil Díaz, L. M. Establishment and characterization of a competitive exclusion bacterial culture derived from Nile tilapia (Oreochromis niloticus) gut microbiomes showing antibacterial activity against pathogenic Streptococcus agalactiae. PLOS ONE. (2019); 14(5), e0215375. https://doi.org/10.1371/JOURNAL.PONE.0215375

Mohammadian T, Nasirpour M, Tabandeh MR, Heidary AA, GhaneiMotlagh R, Hosseini SS. Administrations of autochthonous probiotics altered juvenile rainbow trout Oncorhynchus mykiss health status, growth performance and resistance to Lactococcus garvieae, an experimental infection. Fish Shellfish Immunol (2019). 86:269–79. doi: 10.1016/ j.fsi.2018.11.052

Nayak SK. Probiotics and immunity: a fish perspective. Fish Shellfish Immunol (2010). 29(1):2–14. doi: 10.1016/j.fsi.2010.02.017

Pirarat N, Pinpimai K, Endo M, Katagiri T, Ponpornpisit A, Chansue N, et al. Modulation of intestinal morphology and immunity in Nile tilapia (Oreochromis niloticus) by Lactobacillus rhamnosus GG. Res Vet Sci (2011). 91(3):e92–7. doi: 10.1016/j.rvsc.2011.02.014

Picchietti S, Mazzini M, Taddei AR, Renna R, Fausto AM, Mulero V, et al. Effects of administration of probiotic strains on GALT of larval gilthead seabream: Immunohistochemical and ultrastructural studies. Fish Shellfish Immunol (2007). 22(1):57–67. doi: 10.1016/j.fsi.2006.03.009

Picchietti S, Fausto AM, Randelli E, Carnevali O, Taddei AR, Buonocore F, et al. Early treatment with Lactobacillus delbrueckii strain induces an increase in intestinal T-cells and granulocytes and modulates immune-related genes of larval Dicentrarchus labrax (L.). Fish Shellfish Immunol (2009). 26(3):368– 76. doi: 10.1016/j.fsi.2008.10.008

Ringø E, Van Doan H, Lee SH, Soltani M, Hoseinifar SH, Harikrishnan R, et al. Probiotics, lactic acid bacteria and bacilli: interesting supplementation for aquaculture. J Appl Microbiol (2020). 129(1):116–36. doi: 10.1111/jam.14628

Rombout JHWM, Abelli L, Picchietti S, Scapigliati G, Kiron V. Teleost intestinal immunology. Fish Shellfish Immunol (2011). 31(5):616–26. doi: 10.1016/j.fsi.2010.09.001 107.

Salinas I, Abelli L, Bertoni F, Picchietti S, Roque A, Furones D, et al. Monospecies and multispecies probiotic formulations produce different systemic and local immunostimulatory effects in the gilthead seabream (Sparus aurata L.). Fish Shellfish Immunol (2008). 25(1):114–23. doi: 10.1016/j.fsi.2008.03.011

Salinas I, Zhang Y-A, Sunyer JO. Mucosal immunoglobulins and B cells of teleost fish. Dev Comp Immunol (2011). 35(12):1346–65. doi: 10.1016/ j.dci.2011.11.009 106.

Salinas I, Parra D. Fish mucosal immunity: intestine. In: Mucosal Health in Aquaculture. (2015). Cambridge, Massachusetts (USA): Academic Press p. 135–70. doi: 10.1016/B978-0-12-417186-2.00006-6

Shabirah A, Rosidah R, Mulyani Y, Lili W. Effect of types isolated lactic acid bacteria on hematocrit and differential leukocytes fingerling common carp (Cyprinus carpio L.) infected with Aeromonas hydrophila bacteria. World News Nat Sci (2019). 24:22–35.

Sharifuzzaman S, Al-Harbi A, Austin B. Characteristics of growth, digestive system functionality, and stress factors of rainbow trout fed probiotics Kocuria SM1 and Rhodococcus SM2. Aquaculture (2014). 418:55–61. doi: 10.1016/j.aquaculture.2013.10.006

Soltani M, Ghosh K, Hoseinifar SH, Kumar V, Lymbery AJ, Roy S, et al. Genus Bacillus, promising probiotics in aquaculture: aquatic animal origin, bio-active components, bioremediation and efficacy in fish and shellfish. Rev Fish Sci Aquac (2019). 27(3):331–79. doi: 10.1080/23308249.2019.1597010

Soltani M, Kane A, Taheri-Mirghaed A, Pakzad K, Hosseini-Shekarabi P. Effect of the probiotic, Lactobacillus plantarum on growth performance and haematological indices of rainbow trout (Oncorhynchus mykiss) immunized with bivalent streptococcosis/lactococcosis vaccine. Iran J Fish Sci (2019). 18 (2):283–95. doi: 10.22092/ijfs.2018.117757

Van Doan H, Hoseinifar SH, Tapingkae W, Seel-Audom M, Jaturasitha S, Dawood MA, et al. Boosted growth performance, mucosal and serum immunity, and disease resistance Nile tilapia (Oreochromis niloticus) fingerlings using corncob-derived xylooligosaccharide and Lactobacillus plantarum CR1T5. Probiotics Antimicrob Proteins (2020). 12(2):400–11. doi: 10.1007/s12602-019-09554-5

Xia Y, Lu M, Chen G, Cao J, Gao F, Wang M, et al. Effects of dietary Lactobacillus rhamnosus JCM1136 and Lactococcus lactis subsp. lactis JCM5805 on the growth,

intestinal microbiota, morphology, immune response and disease resistance of juvenile Nile tilapia, Oreochromis niloticus. Fish Shellfish Immunol (2018). 76:368–79. doi: 10.1016/ j.fsi.2018.03.020

Xiao, F. · Liao, L. · Xu, Q. ... Host-Microbiota interactions and responses to grass carp reovirus infection in Ctenopharyngodon idellus Environ Microbiol (2021); 23:431-447

Zhang DX, Kang YH, Zhan S, Zhao ZL, Jin SN, Chen C, et al. Effect of Bacillus velezensis on Aeromonas veronii-induced intestinal mucosal barrier function damage and inflammation in crucian carp (Carassius auratus). Front Microbiol (2019). 15:2663(10). doi: 10.3389/fmicb.2019.02663

Zhang, Y., Qi, X., Zhang, Z., Jin, Z., Wang, G., & Ling, F. Effects of dietary Cetobacterium somerae on the intestinal health, immune parameters and resistance against Nocardia seriolae of largemouth bass, Micropterus salmoides. Fish & shellfish immunology (2023). 135, 108693. https://doi.org/10.1016/j.fsi.2023.108693

Response to Reviewer 3 Comments

Comment 1: “However, I have significant concerns regarding the overall presentation and writing style of the manuscript. It does not adhere to the formatting and structural guidelines typically expected for scientific manuscripts, such as those outlined by PLOS ONE. The writing style resembles that of a paper prepared for a different discipline, rather than aligning with standard scientific conventions. Additionally, the English language requires substantial editing for clarity, grammar, and readability to meet the standards of a peer-reviewed journal.

Response 1: The authors have revised the manuscript according to the reviewers' comments, and the quality is improved.

Comment 2: In the Materials and Methods section, please provide detailed information on how Cetobacterium sp. nov C33 was cultured under anaerobic conditions, including the specific broth medium used and any relevant growth parameters.

Response 2: The methodology was clarified as follows (Lines 148-151, 207-229)

Commens 3: To improve transparency and enhance the manuscript's clarity for readers in the field, I recommend rewriting sentences like: 'The gut-associated microbiota modulation was studied using culture independent techniques by 16S amplicons Illumina sequencing.' As currently phrased, it is difficult to understand. Consider rewording in a clear and concise manner, such as: 'The composition of gut-associated microbiota was analyzed using culture-independent 16S rRNA gene sequencing via Illumina technology.' This helps ensure that the methods and findings are easily understood and accessible to a broader scientific audience.

Response 3: We rewrite in lines 175-176 “The composition of gut-associated microbiota was analyzed using culture-independent 16S rRNA gene sequencing via Illumina technology”.

Comments 4: To improve transparency and ensure a clearer understanding, please clarify how microbial diversity was calculated and how the data were normalized. The statement, 'To estimate microbial diversity, abundance tables were normalized with Total Sum Scaling (TSS) in each sample,' lacks sufficient detail. It would be helpful to specify the exact steps used in the normalization process, including how TSS was applied and whether diversity metrics were calculated on raw or normalized data. Providing this information will strengthen reproducibility and comprehension for readers.

Response 4: We clarify in Lines 207-235: “The starting point was a data table where each row represents a sample and each column an OTU (operational taxonomic

unit). The values in the table are the read counts of each OTU in each sample. Data filtering and normalization were performed to remove low-quality or uninformative features from the raw abundance data, improving subsequent statistical analysis. Outliers in the abundance data were identified using interquartile range (IQR)-based filtering, which is robust in handling skewed microbiome data and minimizes the influence of extreme values that can distort the overall analysis. Data filtering and normalization were performed to remove low-quality or uninformative features from the raw abundance of data, improving subsequent statistical analysis (Zhou *et al*., 2023). Features with very low counts (fewer than 4 reads in less than 20% of samples) were filtered out to exclude rare taxa, focusing the analysis on microbes with consistent presence and biological relevance (Cao *et al*., 2021; Cameron *et al*., 2021). A low-variance filter, using RIC variances, was applied to remove features with low variability between samples, ensuring robustness in subsequent statistical analyses. Normalization addressed variability in sampling depth and data sparsity, and values were normalized by dividing the number of reads for each OTU by the total number of reads in each sample, converting counts to proportions (Cappellato *et al*., 2022). Each value in the table represents the proportion of reads for a specific OTU relative to the total reads in the sample. Once the data were normalized with TSS, Alpha diversity indices were calculated, such as the measure of observed species richness with Chao1 index, and species diversity with ASVs the Shannon index measure".

Response to Reviewer 4 Comments

Comment 1: The abstract could be a little larger and elaborate.

Response 1: The abstract was improved.

Comments 2: The overall writing is average not up to the mark. It needs more grammartical refining.

Response 2: Thanks for pointing this out. We have revised the entire manuscript to meet the publication standard according to the suggestions.

Comment 3: More references should be used to validate the given informations properly.

Response 3: Thanks for pointing this out. We have revised the entire manuscript and include following references:

9. Melo-Bolívar, J. F., Ruiz Pardo, R. Y., Quintanilla-Carvajal, M. X., Díaz, L. E., Alzate, J. F., Junca, H., Rodríguez Orjuela, J. A., & Villamil Diaz, L. M. (2023). Evaluation of dietary single probiotic isolates and probiotic multistrain consortia in growth performance, gut histology, gut microbiota, immune regulation, and infection resistance of Nile tilapia, Oreochromis niloticus, shows superior monostrain performance. Fish & Shellfish Immunology, 140, 108928. https://doi.org/10.1016/J.FSI.2023.108928

19. FA, R. (2018). Genome-Nutrifortified Diets-Their Disease Protection and Remedy Potential. Journal of Probiotics & Health, 06(02). https://doi.org/10.4172/2329-8901.1000204

22. Kilkenny, C., Browne, W. J., Cuthill, I. C., Emerson, M., & Altman, D. G. Improving bioscience research reporting: the ARRIVE guidelines for reporting animal research. PLoS biology. (2010; 8(6), e1000412. https://doi.org/10.1371/journal.pbio.1000412

23. Rose, M., Everitt, J., Hedrich, H., Schofield, J., Dennis, M., Scott, E., & Griffin, G. (2013). ICLAS working group on harmonization: International guidance concerning the production care and use of genetically-altered animals. Laboratory Animals, 47(3), 146–152. https://doi.org/10.1177/0023677213479338

25. Sharifuzzaman, S. M., & Austin, B. (2010). Development of protection in rainbow trout (Oncorhynchus mykiss, Walbaum) to Vibrio anguillarum following use of the probiotic Kocuria SM1. Fish & Shellfish Immunology, 29(2), 212–216. https://doi.org/10.1016/J.FSI.2010.03.008

31. Zhou, R., Ng, S. K., Sung, J. J. Y., Goh, W. W. bin, & Wong, S. H. (2023). Data pre-processing for analyzing microbiome data – A mini review. Computational and

Structural Biotechnology Journal, 21, 4804–4815. https://doi.org/10.1016/J.CSBJ.2023.10.001

32. Cao, Q. X., Sun, X., Rajesh, K., Chalasani, N., Gelow, K., Katz, B., Shah, V. H., Sanyal, A. J., & Smirnova, E. (2020). Effects of microbiome rare taxa filtering on statistical analysis. https://doi.org/10.21203/RS.3.RS-34781/V1

33. Cameron, E. S., Schmidt, P. J., Tremblay, B. J. M., Emelko, M. B., & Müller, K. M. (2021). Enhancing diversity analysis by repeatedly rarefying next generation sequencing data describing microbial communities. Scientific Reports, 11(1). https://doi.org/10.1038/S41598-021-01636-1

34. Cappellato, M., Baruzzo, G., & Camillo, B. di. (2022). Investigating differential abundance methods in microbiome data: A benchmark study. PLoS Computational Biology, 18(9), e1010467. https://doi.org/10.1371/JOURNAL.PCBI.1010467
