# Supplementary material for: Anaerobic bacteria Cetobacterium sp. nov C33 plays a crucial role in the intestinal microbial balance and regulation of gene expression to immune and metabolic responses in Nile tilapia
Source: PLoS One. 2026 May 7;21(5):e0344851. doi: 10.1371/journal.pone.0344851 (PMC13152124; doi:10.1371/journal.pone.0344851)
Supplement: Attachment — Submitted filename: Plos One Peer Review Comments.docx [file pone.0344851.s011.docx]

**Peer Reviewer s Comments**

The Paper entitled Anaerobic bacteria *Cetobacterium sp. nov C33* plays a crucial role in the intestinal microbial balance and regulation of gene expression to immune and metabolic responses in Nile Tilapia which has been submitted to publish in *PLOS One* presents a good investigative efforts but the hypothesis derived from the obtained data is scientifically and logically wrong very likely because there is a gap in present understanding on knowledge.

Low cell population e.g.10^8^cells/g of Anaerobic bacteria *Cetobacterium sp. nov C33* present in feed are practically too less to enable the fed innoculum to appear as dominating species in Nile Tilapia particularly in the case when it requires anaerobic condition to grow at 28C whereas the temperature of fish body is usually lower than 28C with limited availability of oxygen. However these cells by virtue of their high hydrophobicity can make clusters having potential to adhere with cells membrane of cells lining the gut effecting Oxygen, cholesterol and different metallic ions transport particularly Copper and Iron across the cell membranes effecting the host cells functions of target cells and also interconnected cells translating the impact across the organs connected through body systems modulating genes network operations by virtue of copper and iron balance in associated and dissociated forms effected differently by oxygen availability at cellular level, enabling cells to attain new phenotypes with altered homeostasis supported environment leading to the shift in microbial populations and shift in body cell functions over the period attaining altered normal. In response of stress conditions in gut the anaerobic bacteria *Cetobacterium sp. nov C33* produce pigments and other secondary metabolites e.g. chelating agents enzymes etc which alters the iron availability and its accessibility in the cell environment which is is evident by up regulation and down regulation of different genes without activating an inflammatory pathway inspite of involving immune system of the host and without uncontrolled fluctuation in energy demand in body cells subsets.

Using yeast as a model, it has already been established that *CTR1*,(*CTR1* gene encodes high affinity copper transporter) *SOD1,* superoxide dismutase 1 gene, GSH encoding genes and cholesterol cellular biosynthesis genes which are interdependently co-regulated at transcriptional level to sustain metallic ions homeostasis, energy homeostasis, cell s aging leading to cell evolution or cell death. Papers revealing the underlying molecular mechanisms have already been submitted.

The work presented in the paper seems to be very exciting but the hypothesis drawn is wrong very likely because of the gap in the knowledge.

I would recommend publishing this piece of work in *PLOS one* after revising the hypothesis presented in the paper on the ground mentioned above after making through independent critical analysis of published data and my published and unpublished but submitted work which can be obtained from Professor Simon Avery University of Nottingham United Kingdom by contacting him on e-mail [Simon.Avery@nottingham.ac.uk](mailto:Simon.Avery@nottingham.ac.uk) if he kindly agrees to do so.

**Helpful References**

**1)** Bishop, A.L, Rab, F.A, Sumner, E.R, Avery, S.V (2007). Phenotypic heterogeneity can enhance rare-cell survival in 'stress-sensitive' yeast populations. *Mol Microbiol.* 63(2):507-20. doi: 10.1111/j.1365-2958.2006.05504.x. Epub 2006 Dec 14. PMID: 17176259

**2)** Rab, F.A. Solving a biological problem: Research methodologyVDM Verlag Dr. Müller (April 7, 2010) ISBN-10 ‏ : ‎ 9783639247497 ISBN-13 ‏ : ‎ 978-3639247497

**3)** Rab, F.A.(2014)Environmentally Modulated Evolution through Genetic Regulation Information Systems for Biotechnology *ISB News* *Reports* June/July

**4)** Rab, F.A.(2015) A Global Prospective on Research. *Academe* September October

**5)** Rab, F.A.(2016) Biotechnology and its Potentials and Challenges
*EC Agriculture* 3.4 : 705-707

**6**)Rab, F.A.(2017)(a)Who Should Come in Research? *EC* *Nutrition* 6*.*3:102-104

**7)** Rab, F.A. (2017)(b) Eat Fresh Live Young *EC* *Nutrition* RCO.01:03-05

**8)** Rab, F.A(2018)(a) Is Sugar an Accessory or a Neccessary.*EC Nutrition* 13.4:236-237

**9)** Rab, F.A.(2018)(b)Drug Disease Relationship and the Role of Food in Healthy Living.*EC Nutrition* 13.8 doing the bold one

**10)** Rab, F.A.(2018)(c)Genome-Nutrifortified Diets-Their Disease Protection and Remedy Potential. *J*.*Prob* *Health* 6 :204 doi: 10.4172/2329-8901.1000204

Rab FA (2018) Genome-Nutrifortified Diets-Their Disease Protection and Remedy Potential. J Prob Health 6: 204. doi:

10.4172/2329-8901.1000

Rab FA (2018) Genome-Nutrifortified Diets-Their Disease Protection and Remedy Potential. J Prob Health 6: 204. doi:

10.4172/2329-8901.1000

**11)** Rab, F.A.(2019)(a) Comparison between Safety Risks Associated with Domestically Processed Food and Commercially Manufactured Processed Food across the Food Supply Chain.*EC Nutrition* 14.5:414-416.

**12)** Rab, F.A(2019)(b) Is Hunger More Dangerous than having Mal-Nutrition or Consuming Unsafe Diet.*EC Nutrition* 14.12:01-05

**13)** Rab, F.A.(2020)(a) Hurdles in Progression of Knowledge and its Global Impact. *Glob J Res Rev* Vol.7 No.1:47. DOI: 10.36648/2393-8854.7.1.47

**14)** Rab, F.A. (2020)(b) Halal or Haram-New for Religious Scholars Muslim World and Food Supply Chain Stake Holders. *Int J Nutr Sci & Food Tech*. 6:3

**15)** Rab, F.A. (2021) Food items biologically tailored to meet nutritional deficiency challenge during Covid 19 Pandemic. *J Prob Health*. 9:233.

**16)** Abdur Rab, F., Hassan, A. (2022). Tourism, Health Promoting Food Domain and Technology Applications: Individual’s Genes Reservoir, Environmental Change and Food in Natural Health Context. In: Hassan, A. (eds) Handbook of Technology Application in Tourism in Asia. Springer, Singapore. <https://doi.org/10.1007/978-981-16-2210-6_53>

**17)** Rab et al.,(Co authored with Dr Sami Farooq, Dean of School of Management Sciences, Ghulam Ishaq Khan Institute of Engineering Sciences and Technology Swabi Pakistan) (2023) COVID 19- Technology driven Economically coordinated Supply Chain Chaos, its Remedy and Recommendations [Complete Paper and Science Policy Brief had been discussed in World Health Organization (WHO) s Meeting held on 4^th^ May 2023 and in United Nation (UN) s Science Forum Meeting ( Science Technology and Innovation Forum e.g. STI Forum) held on 3-4 May 2023 in United States of America (USA) on the author (Dr Faiza Abdur Rab) s request which she made in capacity of Reviewer for all the Research Journals being published by The Royal Society of Chemistry. These papers are available on request].

**18)**Rab, F.A.(2023) Would Science Knowledge, Food and Agriculture Sector be Considered Basic Human Services or Commercial Services in this Post Covid19 Era? Decision will Determine the Destination. *EC Nutrition* 18.10: 01-08

**19)** Rab, F.A. (2023) Corona Virus (**SARS-CoV-2 )** Rewinding Models in Science and Business and Redefining Illness Manifestations **[submitted in Kuwait Journal of Science by Elsevier]**

**(20)** Rab F.A (2023) Covid 19 Virus- Genomic and Molecular Gaming and the Role of Diet Preparations in Therapy **[ under review in one of the well reputed Journals by Springer Nature]**

**21) Rab, F.A. (2024)** Universal Evolutionary capacitor switch operation regulation gimmicks driving illness and natural cure **[ under process in one of the well reputed Journals by Nature]**

**22)** Colorado Gómez, M. A., Melo-Bolívar, J. F., Ruíz Pardo, R. Y., Rodriguez, J. A., & Villamil, L. M. (2023). Unveiling the Probiotic Potential of the Anaerobic Bacterium *Cetobacterium* sp. nov. C33 for Enhancing Nile Tilapia (*Oreochromis niloticus*) Cultures. Microorganisms, 11(12), 2922. <https://doi.org/10.3390/microorganisms11122922>

**23)** Chatfield, C. H., & Cianciotto, N. P. (2007). The secreted pyomelanin pigment of Legionella pneumophila confers ferric reductase activity. *Infection and immunity*, *75*(8), 4062–4070. <https://doi.org/10.1128/IAI.00489-07>

**24)** Maret W.(2024) The Extracellular Metallometabolome: Metallophores, Metal Ionophores, and Other Chelating Agents as Natural Products. *Natural Product Communications*.;19(8). doi:[10.1177/1934578X241271701](https://doi.org/10.1177/1934578X241271701)
