# Supplementary material for: Anaerobic bacteria Cetobacterium sp. nov C33 plays a crucial role in the intestinal microbial balance and regulation of gene expression to immune and metabolic responses in Nile tilapia
Source: PLoS One. 2026 May 7;21(5):e0344851. doi: 10.1371/journal.pone.0344851 (PMC13152124; doi:10.1371/journal.pone.0344851)
Supplement: Attachment — Submitted filename: PLos One Peer Review comments for Revision one.docx [file pone.0344851.s013.docx]

**Reviewer s comments**

The comments of the authors in response of the points I raised have failed to justify the given scientific status of the revised paper.

For instance technically speaking in a common person s language hypothesis is referred as a possibility. Merely substituting the synonyms cannot serve the purpose to make a scientific content authentic

The authors failed to present a scientifically authentic counter argument against my stance " Low cell population e.g.108 cells/g of Anaerobic bacteria *Cetobacterium* sp. nov C33 present in feed are practically too less to enable the fed inoculums to appear as dominating species in Nile Tilapia particularly in the case when it requires anaerobic condition to grow at 28°C whereas the temperature of fish body is usually lower than 28°C with limited availability of oxygen ".

Furthermore it seems that paper under revision has been written with inadequate literature search reflected as deficient understanding of relevant domains of knowledge. There is no single living entity which does operate without interplay of mineral ions. As far as production of pigments are concerned, genes encoding them may not be present on genome of the bacteria such as they can be present on plasmid or production of pigments can be an induced manifestation of a biological event or change in the environment.

**Supporting scientific abstract statement**

"microorganisms might evolve responses to sublethal concentrations of these metabolites, either to protect themselves from inhibition or to change certain behaviors in response to the local abundance of another species. Here, we report that violacein production by C. violaceum ATCC 31532 is induced in response to hygromycin A from Streptomyces sp. 2AW, and we show that this response is dependent on inhibition of translational polypeptide elongation and a previously uncharacterized two-component regulatory system. The breadth of the transcriptional response beyond violacein induction suggests a surprisingly complex metabolite-mediated microbe-microbe interaction and supports the hypothesis that antibiotics evolved as signal molecules".

**Reference**

Lozano, G. L., Guan, C., Cao, Y., Borlee, B. R., Broderick, N. A., Stabb, E. V., & Handelsman, J. (2020). A Chemical Counterpunch: Chromobacterium violaceum ATCC 31532 Produces Violacein in Response to Translation-Inhibiting Antibiotics. *mBio*, *11*(3), e00948-20. <https://doi.org/10.1128/mBio.00948-20>.

The concerns raised on assessing the submission entitled Anaerobic bacteria *Cetobacterium* sp. nov C33 plays a crucial role in the intestinal microbial balance and regulation of gene expression to immune and metabolic responses in Nile Tilapia have not been addressed while revising the paper, scientific flaws still sustain in the revision, that builds a ground to recommend major revision of the revised version of the submission while addressing the points raised by me in capacity of reviewer prior publishing the paper.
