# Supplementary material for: Anaerobic bacteria Cetobacterium sp. nov C33 plays a crucial role in the intestinal microbial balance and regulation of gene expression to immune and metabolic responses in Nile tilapia
Source: PLoS One. 2026 May 7;21(5):e0344851. doi: 10.1371/journal.pone.0344851 (PMC13152124; doi:10.1371/journal.pone.0344851)
Supplement: Attachment — Submitted filename: Response Letter 30 oct.docx [file pone.0344851.s014.docx]

October 31, 2025

Dr. Amel Mohamed El Asely

Academic Editor

*PLOS ONE*

**Subject: Revised Letter to the Editor**

Dear Dr. El Asely,

We sincerely appreciate your consideration of our manuscript **PONE-D-25-18938**, entitled “The anaerobic bacterium Cetobacterium sp. nov. C33 plays a crucial role in intestinal microbial balance and the regulation of gene expression for immune and metabolic responses in Nile tilapia.”

We sincerely thank you and the reviewers for the time, effort, and valuable insights provided in the evaluation of our manuscript. We have carefully considered and addressed all comments, which have greatly contributed to enhancing the clarity, rigor, and overall quality of the revised version.

Please find enclosed a detailed, point-by-point response to the reviewers’ observations, as well as the revised manuscript for your consideration. We hope that the revisions satisfactorily meet the expectations of the reviewers and the editorial board.

Thank you once again for your time, insightful comments, and continued consideration of our work.

With kind regards,

**Luisa Villamil**
(on behalf of all co-authors)
Universidad de La Sabana, Colombia

**Journal Requirements:**

**Comment 1:** If the reviewer comments include a recommendation to cite specific previously published works, please review and evaluate these publications to determine whether they are relevant and should be cited. There is no requirement to cite these works unless the editor has indicated otherwise.

**Response 1:** We sincerely appreciate the editor’s guidance and the reviewer’s thoughtful efforts to improve our manuscript. Regarding Reviewer 2, the suggestion to include a list of 26 additional publications, we carefully reviewed each of them to evaluate their relevance to our study. Only one of these references was found to be directly related to the topic addressed in our manuscript and has therefore been included: “FA, R. (2018). Genome-Nutrifortified Diets-Their Disease Protection and Remedy Potential. Journal of Probiotics & Health, 06(02). https://doi.org/10.4172/2329-8901.1000204”.

We noticed that most of the suggested papers were authored or co-authored by the reviewer and focus on research areas that differ from the central objectives of our study. We mention this respectfully and only to clarify that our decision was based solely on scientific relevance and not on the origin of the suggested works. We believe that maintaining references closely aligned with the study’s scope ensures coherence and accuracy in the scientific discussion.

**Reviewers' comments:**

**Reviewer's Responses to Questions**

**Comments to the Author**

**Comment 1.** If the authors have adequately addressed your comments raised in a previous round of review and you feel that this manuscript is now acceptable for publication, you may indicate that here to bypass the “Comments to the Author” section, enter your conflict of interest statement in the “Confidential to Editor” section, and submit your "Accept" recommendation.

Reviewer #2: (No Response)

**Comment 2.** Is the manuscript technically sound, and do the data support the conclusions?

The manuscript must describe a technically sound piece of scientific research with data that supports the conclusions. Experiments must have been conducted rigorously, with appropriate controls, replication, and sample sizes. The conclusions must be drawn appropriately based on the data presented.

Reviewer #2: Partly

**Response 2:** We sincerely thank the reviewer for emphasizing the importance of methodological rigor and data reliability. We fully agree that conclusions must be supported by robust and reproducible evidence. In this regard, we would like to clarify that all experiments in the submitted manuscript were conducted following rigorous scientific standards, including appropriate controls, biological replicates, and statistically adequate sample sizes.

The results presented were carefully analyzed, and the conclusions are directly supported by the data obtained. Although Reviewer 2 mentioned that these criteria were “partially fulfilled,” we respectfully consider that the current version of the manuscript already meets these requirements in full. The reviewer’s comment did not specify a particular aspect requiring improvement; however, we have re-examined the methodology and data presentation to ensure clarity and confirm the reproducibility and soundness of the findings.

**Comment 3.** Has the statistical analysis been performed appropriately and rigorously?

Reviewer #2: No

**Response 3:** We appreciate the reviewer’s observation regarding the statistical analyses. Statistical analyses were indeed performed and described in the submitted version of the manuscript; however, we have now improved the methodological description to provide greater clarity and transparency.

Specifically, we have included detailed information in the revised Methods section regarding the statistical procedures applied. In addition, Tables 1 and 2 now present the statistical data supporting the significant differences observed among bacterial genera. Supplementary Tables S1 and S2 have also been updated to include the statistical parameters for the differentially upregulated and downregulated genes, which were analyzed in R using the **edgeR** pipeline. These additions aim to further demonstrate that the analyses were conducted appropriately and rigorously.

**Comment 4.** Have the authors made all data underlying the findings in their manuscript fully available?

The PLOS Data policy requires authors to make all data underlying the findings described in their manuscript fully available without restriction, with rare exception (please refer to the Data Availability Statement in the manuscript PDF file). The data should be provided as part of the manuscript or its supporting information, or deposited to a public repository. For example, in addition to summary statistics, the data points behind means, medians and variance measures should be available. If there are restrictions on publicly sharing data—e.g. participant privacy or use of data from a third party—those must be specified.

Reviewer #2: No

**Response 4**: We Uploaded all data as supplementary information.

Metataxonomic data:

Files: ASV_Ceto, Meta_Ceto, Taxa_Ceto, Data Metataxonomy, Summary_file

Transcriptomics data:

Files: Data, Desing, Metadata RNAseq

Additionally, the results of the sequence file we upload to SRA NCBI

| **Bioproject** | **Title** | **BioSample** | **SRA** |
| --- | --- | --- | --- |
| [PRJNA1306746](https://dataview.ncbi.nlm.nih.gov/object/PRJNA1306746) | 16S rRNA gene amplicons of fish gut microbiomes Metagenome | [SAMN50644908](https://dataview.ncbi.nlm.nih.gov/object/59717289) | [7](https://dataview.ncbi.nlm.nih.gov/objects?linked_to_id=PRJNA1306746&archive=sra) |
| [PRJNA1306769](https://dataview.ncbi.nlm.nih.gov/object/PRJNA1306769) | fish head kidney transcriptomic datasets Transcriptome | [SAMN50645431](https://dataview.ncbi.nlm.nih.gov/object/59718184) | [7](https://dataview.ncbi.nlm.nih.gov/objects?linked_to_id=PRJNA1306769&archive=sra) |

Also, the data will be available in DOI:10.5281/zenodo.17479129 or our OneDrive folder if reviewers request it. Please request access to the OneDrive repository by emailing [luisa.villamil@unisabana.edu.co](mailto:luisa.villamil@unisabana.edu.co)

Metataxonomic data:

| Sample | Treatment | File | File | Sample ID |
| --- | --- | --- | --- | --- |
| C7 | Control | C7_1.fq.gz | C7_2.fq.gz | MCD1 |
| C8 | Control | C8_1.fq.gz | C8_2.fq.gz | MCD2 |
| C9 | Control | C9_1.fq.gz | C9_2.fq.gz | MCD3 |
| D2 | *Cetobacterium* | D2_1.fq.gz | D2_2.fq.gz | MC33D1 |
| D3 | *Cetobacterium* | D3_1.fq.gz | D3_2.fq.gz | MC33D2 |
| D4 | *Cetobacterium* | D4_1.fq.gz | D4_2.fq.gz | MC33D3 |
| D5 | *Cetobacterium* | D5_1.fq.gz | D5_2.fq.gz | MC33D4 |

Transcriptomic data:

| Sample | Treatment | File | File | Sample ID |
| --- | --- | --- | --- | --- |
| R42 | Control | R42_1.fq.gz | R42_2.fq.gz | TCD1 |
| R47 | Control | R47_1.fq.gz | R47_2.fq.gz | TCD2 |
| R53 | Control | R53_1.fq.gz | R53_2.fq.gz | TCD3 |
| R57 | *Cetobacterium* | R57_1.fq.gz | R57_2.fq.gz | TC33D1 |
| R58 | *Cetobacterium* | R58_1.fq.gz | R58_2.fq.gz | TC33D2 |
| R59 | *Cetobacterium* | R59_1.fq.gz | R59_2.fq.gz | TC33D3 |
| R60 | *Cetobacterium* | R60_1.fq.gz | R60_2.fq.gz | TC33D4 |

**Comment 5.** Is the manuscript presented in an intelligible fashion and written in standard English?

PLOS ONE does not copyedit accepted manuscripts, so the language in submitted articles must be clear, correct, and unambiguous. Any typographical or grammatical errors should be corrected at revision, so please note any specific errors here.

Reviewer #2: Yes

**Response 5**. We have worked in the improved version.

**Comment 6. Review Comments to the Author**

Reviewer #2: Reviewer s comments

**Comment 1**: The comments of the authors in response of the points I raised have failed to justify the given scientific status of the revised paper.

For instance, technically speaking in a common person’s language hypothesis is referred as a possibility. Merely substituting the synonyms cannot serve the purpose to make a scientific content authentic.

The authors failed to present a scientifically authentic counter argument against my stance " Low cell population e.g.10^8^ cells/g of Anaerobic bacteria *Cetobacterium* sp. nov C33 present in feed are practically too less to enable the fed inoculums to appear as dominating species in Nile Tilapia particularly in the case when it requires anaerobic condition to grow at 28°C whereas the temperature of fish body is usually lower than 28°C with limited availability of oxygen ".

**Response 1:** We fully agree that scientific terminology must be used with precision and consistency to ensure clarity and authenticity. Accordingly, we have carefully reviewed the wording throughout the manuscript to verify that all terms are scientifically accurate and used in their correct context.

We thank the reviewer for the opportunity to clarify this point. The increase of Cetobacterium observed in our study is an empirical result supported by robust metabarcoding data, not a conjecture. The 16S rRNA gene amplicon analyses demonstrated a clear and statistically supported shift in Cetobacterium relative abundance within the intestinal bacterial community following treatment. These results are fully detailed in the Results section and in the revised Supplementary Tables. Furthermore, the isolate Cetobacterium sp. nov. C33 was obtained from a competitive-exclusion culture (CFCEC) derived from tilapia intestinal content, following protocols previously established by our group (Melo-Bolívar *et al*., 2019). The origin of this isolate from tilapia gut material provides a strong biological rationale for its ability to persist and thrive in the tilapia gastrointestinal environment. Overall, the enrichment of Cetobacterium in our data represents a reproducible, data-driven finding consistent across both metabarcoding and culture-based analyses. Our experimental design, probiotic dose, and interpretation are aligned with the current literature, which has now been fully cited in the revised manuscript.

We included Lines 500 – 517 “For probiotic applications in Oreochromis niloticus, several studies have demonstrated positive results using bacterial concentrations between 10⁷ and 10⁸ CFU. For example, Wang *et al*. (2008) administered Enterococcus faecium (1 × 10⁷ CFU/mL) for 40 days, resulting in increased final weight. Similarly, Van Doan *et al*. (2019) supplemented diets with Lactobacillus plantarum (1 × 10⁸ CFU/g) for 84 days, enhancing growth performance, while Sewaka *et al*. (2019) reported that Lactobacillus rhamnosus (1 × 10⁸ CFU/g) administered for two weeks improved intestinal histo-morphological parameters in red tilapia. Although lower concentrations, such as 1.1 × 10⁵ CFU/g of Bacillus subtilis used by Hassaan *et al*. (2018), have also shown beneficial effects, most successful applications in tilapia employ probiotic doses within the 10⁷ to 10⁸ CFU range. Comparable results have been reported in other species. Xie *et al*. (2022a) conducted an eight-week trial with 300 common carp (Cyprinus carpio) fed a basal diet supplemented with 2, 3, 4, or 5 g/kg of C. somerae XMX-1 (10⁸ CFU/mL), demonstrating significant improvements in intestinal and hepatic health. Likewise, Xie *et al*. (2022b) performed a four-week experiment with 240 one-month-old adult zebrafish (initial weight 0.28 ± 0.003 g) fed a basal diet supplemented with 10 g/kg of XMX-1 at the same concentration (10⁸ CFU/mL), obtaining similar enhancements in overall health parameters.

**References**

Hassaan, M.S., Soltan, M.A., Jarmołowicz, S., & Abdo, H.S. (2018). Combined effects of dietary malic acid and *Bacillus subtilis* on growth, gut microbiota and blood parameters of Nile tilapia (*Oreochromis niloticus*). *Aquaculture Nutrition*, 24, 83–93.

Melo-Bolívar, J.F., Ruiz Pardo, R.Y., Hume, M.E., Nisbet, D.J., Rodríguez-Villamizar, F., Alzate, J.F., Junca, H., & Villamil Díaz, L.M. (2019). Establishment and characterization of a competitive exclusion bacterial culture derived from Nile tilapia (*Oreochromis niloticus*) gut microbiomes showing antibacterial activity against pathogenic *Streptococcus agalactiae*. *PLOS ONE*, 14(5), e0215375.

Sewaka, M., Trullas, C., Chotiko, A., Rodkhum, C., Chansue, N., Boonanuntanasam, S., & Pirarat, N. (2019). Efficacy of synbiotic *Jerusalem artichoke* and *Lactobacillus rhamnosus* GG-supplemented diets on growth performance, serum biochemical parameters, intestinal morphology, immune parameters, and protection against *Aeromonas veronii* in juvenile red tilapia (*Oreochromis spp.*). *Fish & Shellfish Immunology*, 86, 260–268.

Van Doan, H., Kurian, A., Hoseinifar, S.H., Sel-audom, M., Jaturasitha, S., Tongsiri, S., & Ringø, E. (2019). Dietary inclusion of orange peel-derived pectin and *Lactobacillus plantarum* for Nile tilapia (*Oreochromis niloticus*) cultured under indoor biofloc systems. *Aquaculture*, 508, 98–105.

Wang, Y.-B., Tian, Z.-Q., Yao, J.-T., & Li, W. (2008). Effect of probiotics *Enterococcus faecium* on tilapia (*Oreochromis niloticus*) growth performance and immune response. *Aquaculture*, 277, 203–207.

Xie, M., Hao, Q., Olsen, R.E., Ringø, E., Yang, Y., Zhang, Z., et al. (2022a). Growth performance, hepatic enzymes, and gut health status of common carp (*Cyprinus carpio*) in response to dietary *Cetobacterium somerae* fermentation product. *Aquaculture Reports*, 23, 101046. <https://doi.org/10.1016/J.AQREP.2022.101046>

Xie, M., Xie, Y., Li, Y., Zhou, W., Zhang, Z., Yang, Y., et al. (2022b). Stabilized fermentation product of *Cetobacterium somerae* improves gut and liver health and antiviral immunity of zebrafish. *Fish & Shellfish Immunology*, 120, 56–66. <https://doi.org/10.1016/J.FSI.2021.11.017>

Zhang, Z., Fan, Z., Yi, M., Liu, Z., Ke, X., Gao, F., et al. (2022). Characterization of the core gut microbiota of Nile tilapia (*Oreochromis niloticus*): Indication of a putative novel *Cetobacterium* species and analysis of its potential function on nutrition. *Archives of Microbiology*, 204(12), 1–12. <https://doi.org/10.1007/s00203-022-03301-1>

Zhou, W., Xie, M., Xie, Y., Liang, H., Li, M., Ran, C., & Zhou, Z. (2022). Effect of dietary supplementation of *Cetobacterium somerae* XMX-1 fermentation product on gut and liver health and resistance against bacterial infection of genetically improved farmed tilapia (GIFT, *Oreochromis niloticus*). *Fish & Shellfish Immunology*, 124, 332–342. <https://doi.org/10.1016/J.FSI.2022.04.019>

**Comment 2:** Furthermore it seems that paper under revision has been written with inadequate literature search reflected as deficient understanding of relevant domains of knowledge. There is no single living entity which does operate without interplay of mineral ions. As far as production of pigments are concerned, genes encoding them may not be present on genome of the bacteria such as they can be present on plasmid or production of pigments can be an induced manifestation of a biological event or change in the environment.

Supporting scientific abstract statement

"microorganisms might evolve responses to sublethal concentrations of these metabolites, either to protect themselves from inhibition or to change certain behaviors in response to the local abundance of another species. Here, we report that violacein production by *C*. *violaceum* ATCC 31532 is induced in response to hygromycin A from *Streptomyces* sp. *2AW*, and we show that this response is dependent on inhibition of translational polypeptide elongation and a previously uncharacterized two-component regulatory system. The breadth of the transcriptional response beyond violacein induction suggests a surprisingly complex metabolite-mediated microbe-microbe interaction and supports the hypothesis that antibiotics evolved as signal molecules".

The concerns raised on assessing the submission entitled Anaerobic bacteria *Cetobacterium* sp. nov C33 plays a crucial role in the intestinal microbial balance and regulation of gene expression to immune and metabolic responses in Nile Tilapia have not been addressed while revising the paper, scientific flaws still sustain in the revision, that builds a ground to recommend major revision of the revised version of the submission while addressing the points raised by me in capacity of reviewer prior publishing the paper.

**Response 2:** We thank the reviewer for the comment and the opportunity to clarify this point. We fully agree that mineral ions and environmental conditions play essential roles in microbial physiology and gene regulation, including pigment production in some species. However, in the present study, we did not assess pigment production, nor does Cetobacterium produce pigments under standard conditions, as it is a non-pigmented, strictly anaerobic bacterium. Therefore, the examples referring to pigment induction or plasmid-encoded pigment genes are not applicable to our study system.

Additionally, we included in the Lines 585 – 600 “*Cetobacterium* sp. nov C33 possesses four genome regions with nonribosomal peptide synthase-like NRPS systems (Colorado Gómez *et al*., 2023) that can produce a wide range of bioactive peptides, including antibiotics and immunosuppressants. Importantly, different classes of secondary metabolites secreted by microorganisms, such as siderophores, biosurfactants, and antibiotics, modulate bacterial interactions (Lozano *et al*., 2020). The concept of “competence sensing” suggests that some microbes might have evolved the ability to detect a danger signal through established stress responses and respond by increasing the production of toxins and antibiotics (Tipping *et al*., 2020). An example of this is *Chromobacterium* species, Gram-negative betaproteobacteria known for the production of violacein, a purple pigment with antimicrobial activity against various Gram-positive bacteria (Santos *et al*., 2025). Further studies in microbial ecology are needed to determine whether *Cetobacterium* sp. nov. C33 engages in subtle forms of chemical competition distinct from those of *Chromobacterium violaceum*. Integrating multi-omics approaches could provide deeper insight into the metabolites, genes, and molecular pathways involved in these interactions.”

References

Colorado Gómez, M. A., Melo-Bolívar, J. F., Ruíz Pardo, R. Y., Rodriguez, J. A., & Villamil, L. M. Unveiling the Probiotic Potential of the Anaerobic Bacterium *Cetobacterium* sp. nov. C33 for Enhancing Nile Tilapia (*Oreochromis niloticus*) Cultures. Microorganisms. 2023; 11(12), 2922. <https://doi.org/10.3390/MICROORGANISMS11122922/S1>

Lozano, G. L., Guan, C., Cao, Y., Borlee, B. R., Broderick, N. A., Stabb, E. V., & Handelsman, J. A Chemical Counterpunch: *Chromobacterium violaceum* ATCC 31532 Produces Violacein in Response to Translation-Inhibiting Antibiotics. mBio, 11(3), e00948-20. <https://doi.org/10.1128/mBio.00948-20>.

Tipping, M. J., & Gibbs, K. A. Biofilms: Managing Stress to Navigate Group Dynamics. Current Biology. 2020; 30(7), R324–R326. <https://doi.org/10.1016/j.cub.2020.02.045>

Santos, C. A., Lima, E. M. F., Miguel, C. M. T. S., & Pinto, U. M. Quantification of Violacein in Chromobacterium violaceum and Its Inhibition by Bioactive Compounds. Journal of Visualized Experiments. 2025; August (222). <https://doi.org/10.3791/68507>
