# Supplementary material for: Anaerobic bacteria Cetobacterium sp. nov C33 plays a crucial role in the intestinal microbial balance and regulation of gene expression to immune and metabolic responses in Nile tilapia
Source: PLoS One. 2026 May 7;21(5):e0344851. doi: 10.1371/journal.pone.0344851 (PMC13152124; doi:10.1371/journal.pone.0344851)
Supplement: Attachment — Submitted filename: Point by point answer .docx [file pone.0344851.s015.docx]

Dr. Amel Mohamed El Asely

Academic Editor

PLOS ONE

Dear Dr. Amel,

On behalf of all authors, I would like to sincerely thank you for your time, dedication, and thoughtful editorial oversight of our manuscript entitled “Anaerobic bacteria Cetobacterium sp. nov. C33 plays a crucial role in the intestinal microbial balance and regulation of gene expression related to immune and metabolic responses in Nile tilapia”. We deeply appreciate the careful and constructive guidance provided throughout this rigorous review process.

We highly value the comprehensive assessment carried out over three rounds of review by six independent reviewers. Their comments and suggestions have significantly strengthened the scientific clarity, methodological transparency, and overall robustness of our study. We are grateful for the opportunity to further refine our work and to provide a detailed, point-by-point response to the remaining comments, to reach a timely and favorable resolution.

Below, we present first a general response and then below, a detailed point by point response to all comments raised by the reviewers, the journal and the Academic Editor. All requested changes have been incorporated into the revised manuscript, with tracked changes clearly indicated. These modifications are further supported by expanded descriptions in the Materials and Methods, Results, Discussion, figures, and supplementary materials.

Regarding the request for additional long-term experiments, we acknowledge and fully appreciate this valuable suggestion. We agree that evaluating longer administration periods, different doses, and additional functional endpoints, including pathogen challenge tests, would provide important complementary insights and align closely with our ongoing and future research plans. However, we respectfully note that conducting new in vivo trials of this nature would require substantial experimental redesign, extended rearing periods, additional ethical approvals, and new funding. Unfortunately, at this advanced stage of the review process, after three rounds of revision and extensive responses to six reviewers, it is not feasible to incorporate new experimental trials within the scope of the current manuscript. We are committed to addressing these aspects in future publications.

Regarding technical soundness and statistical rigor, we reaffirm that the experiments presented were conducted with appropriate controls, replication, and sample sizes, and that the conclusions are fully supported by the data. Statistical analyses were performed using established and widely accepted pipelines. Additional methodological details have been incorporated into the Materials and Methods section, and supplementary tables have been expanded to ensure full transparency and reproducibility.

Specific reviewer comments

All editorial and technical comments related to terminology, figure structure, table titles, subtitles, formatting, capitalization of p values, clarification of experimental units, water quality monitoring, and taxonomic presentation have been carefully addressed. Figures and tables have been reorganized as requested, subtitles have been shortened for clarity, and the Discussion has been refocused to emphasize intestinal microbiota modulation, in direct alignment with the core objectives of the study.

We respectfully hope that the revised version now meets the publication criteria of PLOS ONE and look forward with great appreciation to your kind consideration and a favorable decision.

As requested, we have included both a marked-up version of the manuscript highlighting all changes, labeled “Revised Manuscript with Track Changes”, and a clean version without tracked changes, labeled “Manuscript”.

With sincere regards and gratitude,

Luisa Marcela Villamil Díaz

**DETAILED RESPONSES TO REVIEWERS AND JOURNAL REQUIREMENTS**

**===========================================================**

**Journal Requirements**

Comment 1: Please review your reference list to ensure that it is complete and correct. Response 1: The reference list has been carefully reviewed and verified for completeness, accuracy, and consistency with the citations in the manuscript. Any minor formatting adjustments required by the journal style have been applied accordingly.

Reviewer Responses to Questions

1. Adequacy of previous revisions

Reviewer #5: All comments have been addressed

Reviewer #6: All comments have been addressed

2. Is the manuscript technically sound, and do the data support the conclusions?

Reviewer

#5: Partly

Response: In the revised version of the manuscript, we have provided additional methodological details, clarifications, and supporting information to strengthen transparency and address all points previously raised. We believe these additions further substantiate the technical soundness of the study and the validity of the conclusions drawn from the data.

Reviewer #6: Yes

3. Has the statistical analysis been performed appropriately and rigorously?

Reviewer #5: Yes

Reviewer #6: Yes

4. Have the authors made all data underlying the findings in their manuscript fully available?

Reviewer #5: Yes

Reviewer #6: Yes

5. Is the manuscript presented in an intelligible fashion and written in standard English?

Reviewer #5: Yes

Reviewer #6: Yes

**POINT BY POINT ANSWER TO REVIEWERS**

**===================================**

6. Review Comments to the Author

Reviewer #5

Comment 1:

In this manuscript, the authors investigated the effects of dietary probiotic potential of *Cetobacterium* sp. nov. C33, using 16S rRNA amplicon sequencing, on gut microbiota, and the transcriptomic analysis of the head kidney provided insights into immune system modulation. Further studies should be done for long rearing time (8-10 weeks) using different concentrations of this probiotic to evaluate fish performance and immune system modulation, including resistance against possible pathogenic bacterial infection or other stressors.

Response: We appreciate this constructive and scientifically valuable suggestion. As noted by the reviewer, long-term trials with extended rearing periods, multiple dosages, and pathogen challenge tests would indeed provide complementary and important insights into the functional performance and immunological response of Nile tilapia.

However, it is important to clarify that the present manuscript was designed with a specific and clearly defined scope to evaluate, through an integrated metabarcoding and transcriptomic approach, the short-term effects of a novel anaerobic bacterium *Ceto*bacterium sp. nov. C33 on gut microbiota composition and host gene expression in Nile tilapia fingerlings. Studies combining high-resolution microbiome profiling and head kidney transcriptomics in response to *Cetobacterium* administration are still scarce in tilapia, and we believe this work represents a substantial and novel contribution to the field.

Within this framework, the current dataset provides robust evidence of microbiome modulation and associated changes in immune and metabolism-related gene expression. We therefore consider the study to be scientifically solid, complete within its intended objectives, and appropriate for publication in its current form.

We fully agree that the aspects suggested by the reviewer are highly relevant and will be incorporated into our ongoing and future research projects, which will be addressed in subsequent publications.

Comments 2: L144: Diet preparation.

Response: We have corrected the subtitle to “Diets preparation” in line 142 of the revised manuscript.

Comments 3: L168: The alevins of Nile tilapia should be Nile tilapia juveniles.

Response: We respectfully maintain the use of the term “Nile tilapia fingerlings (<5 g)”. According to Wainaina et al. (2023), fingerlings are newly developed, fragile post-larval fish roughly finger-sized, whereas juveniles (5–10 g) are larger and more robust. Therefore, the term “fingerlings” more accurately reflects the developmental stage of the experimental fish used in this study.

Wainaina, M., Opiyo, M. A., Charo-Karisa, H., Orina, P., & Nyonje, B. (2023). On-Farm Assessment of Different Fingerling Sizes of Nile Tilapia (Oreochromis niloticus) on Growth Performance, Survival, and Yield. Aquaculture Studies, 23(2). https://doi.org/10.4194/AQUAST900

Comments 4: L169: at 5 fish per aquarium with four replicated tanks per experimental treatment.

Response: We have revised the text accordingly to read “at 5 fish per aquarium with four replicated tanks per experimental treatment” in lines 165–166.

Comments 5: L171–L173: : Water quality parameters were monitored daily during the feeding trial, with the following average values.

Response: We have clarified this in lines 168–172 as follows: “Water quality parameters were monitored daily throughout the acclimation and feeding trial. Temperature, dissolved

oxygen, and pH were recorded using a multiparameter sensor (Hanna Instruments HI9829). Water quality was maintained within ranges suitable for Nile tilapia: water temperature 28 °C, dissolved oxygen above 5 mg L⁻¹, oxygen saturation 85%, and pH 7.12.”

Comments 6: L174–L175: Shorten the subtitle to “Modulation of intestine microbiota in Nile tilapia fingerling.”

Response: We revised the subtitle to “Modulation of the immune system in Nile tilapia fingerling” in line 173 to better reflect the content of that section.

Comments 7: L247–L248: Shorten the subtitle to “Modulation of the immune system in Nile tilapia fingerling.”

Response: We revised the subtitle accordingly in line 243.

Comments 8: L301–L302: Shorten the subtitle to “Modulation of intestine microbiota in Nile tilapia fingerling.”

Response: We revised the subtitle accordingly in line 295.

Comments 9: Fig. 1. Should be divided to be two Figs; Fig. 1 for diversity indices and Fig. 2 for the taxonomy of intestinal microbiota. And rewrite the results section accordingly. Fig. 1 for diversity indices and Fig. 2 for the taxonomy of intestinal microbiota. And rewrite the results section accordingly.

Response: We divided the original Figure 1 into two figures: Figure 1 now presents diversity indices (lines 315–324) and Figure 2 presents the taxonomy of intestinal microbiota (lines 339–345). The Results section was rewritten to reflect this change.

Comments 10: Tables. Adjust the title of tables to be more specific such as “Changes in the intestinal microbiota of Nile tilapia fingerlings fed with *Cetobacterium* sp. nov C33 for five days.”

Response: Table 1 is now titled: “Bacterial genera with significant reduction in abundance in the intestinal microbiota of Nile tilapia fingerlings in group C33D” (lines 367–368). Table 2 is now titled: “Bacterial genera with a significant increase in abundance in the intestinal microbiota of Nile tilapia fingerlings in group C33D” (lines 397–398).

Comments 11: What is the difference in titles of Table 1 and Table 2?

Response: Table 1 reports bacterial genera that showed a significant reduction in relative abundance, whereas Table 2 reports bacterial genera that showed a significant increase in relative

abundance in the intestinal microbiota of Nile tilapia fingerlings in the C33D group compared with the Control Diet (CD) group.

Comments 12: L422: Shorten the subtitle to “Modulation of the immune system in Nile tilapia fingerling.”

Response: We revised the subtitle accordingly in line 418.

Comments 13: Data in Tables 1–2 and Figs 3–4 should describe the changes in both treatments. Response: We clarified changes in both treatments in the Results section (lines 370–374 and 400–404). For transcriptomic results, comparisons between C33D and CD are explicitly described in the captions of Figures 4 and 5 (lines 431–444 and 458–471), detailing differentially expressed genes, functional annotations, and up- and down-regulated immune-related transcripts.

Comments 14: Discussion should focus on intestinal microbiota modulation.

Response: We revised the Discussion to prioritize changes in intestinal microbiota. We removed sections focused primarily on fish growth and intestinal histomorphometry and strengthened direct comparisons between control and C33D groups, integrating our findings with relevant previous studies.

Reviewer #6

Comments 1: Change “Nile Tilapia” to “Nile tilapia” throughout the text.

Response: We corrected capitalization to “Nile tilapia” throughout the manuscript, including the title and lines 6 and 42.

Comments 2: P value “P” must be capital and italic.

Response: We formatted all statistical notations as italicized capital “P” in the following lines: 234, 240, 282, 303, 323, 324, 369, 370, 399, 400, 450, 494, 1095, and 1102.
